# sncRNA changes induced by tension in hypertrophic scar: sncRNAs in hypertrophic scar

**DOI:** 10.3724/abbs.2022103

**Published:** 2022-08-05

**Authors:** Chuang Yin, Shixi Zhang, Chen Ya, Chuandong Wang, Yimin Liang, Chen Wang

**Affiliations:** 1 Department of Plastic and Reconstructive Surgery Shanghai Ninth People’s Hospital Shanghai Jiao Tong University School of Medicine Shanghai 200011 China; 2 Department of Radiology Shanghai Ninth People’s Hospital Shanghai Jiao Tong University School of Medicine Shanghai 200011 China; 3 Department of Orthopedic Surgery Xinhua Hospital Affiliated to Shanghai Jiao Tong University School of Medicine Shanghai 200092 China.

Hypertrophic scar (HS) is an abnormal fibroproliferative healing process after skin injury including burn, surgery and folliculitis, and HS is sometimes induced without obvious causation especially in susceptible persons [1]. But the specific HS formation mechanism is still obscure. Several hypotheses have been proposed to explain the pathogenesis of hypertrophic scar, the tension hypothesis seems to be the most persuasive theory. There are several lines of clinical evidence supporting this theory: some sites with high HS incidence rate in the body include the dorsum, the mandible, and the presternal skin [2] where the skin tends to be under high tension; some typical HS on the chest will gradually grow into a butterfly like shape [3] whose long axis is parallel to its direction under traction.

At present, many studies focused on the mechanical forces on fibroblasts. For example, if pulmonary surfactant is destroyed by inhalation of polyhexamethylene guanidine, pulmonary fibroblasts can be stimulated by elevated alveolar surface tension to induce pulmonary fibrosis [4]. Non coding RNAs (ncRNAs), which are transcribed from nonprotein-coding DNAs and play major regulatory roles in the cells [ 5, 6] , include microRNA (miRNA), Piwi-interacting RNA (piRNA), small nucleolar RNA (snoRNA), small nuclear RNA (snRNA) and repeatRNA. Recent studies revealed that these ncRNAs could play a regulatory role beyond their original function [7]. piRNAs were once thought to exert mainly transposon silencing functions, but were recently found to have differences in their expressions between healthy controls and people with different types of cancers and myocardial fibrosis [8]. Recently, snoRNAs have been found to function as miRNAs to regulate genes [9]. Therefore, in this study we examined the expressions of these 4 types of ncRNA in tension-related hypertrophic scars.

The tissue specimen was obtained from an exceptional patient who had various degrees of hyperplasia in the same chest scar ( Supplementary Figure S1) which is a postoperative scar from the classical open thoracotomy. Six months after the surgery, the scar was hypertrophic, reddish in color, painful, and free of infection during healing. Langer’s lines are used to describe the direction of tension on the skin surface [10], while there is a demarcation line of tension at the sternum skin ( Supplementary Figure S2). In our case, a skin crease along the demarcation line separating the proliferative area was observed in the preoperative photographs. Therefore, we think that the tension on both sides of the wound in this patient is the main variable during scar formation. Since the existing animal models cannot perfectly simulate human hypertrophic scar, this specimen is highly valuable.

We performed transcriptome sequencing on skin tissues from HS and normal-tension scar (NTS). A total of 378 upregulated genes versus 365 downregulated genes and 680 upregulated transcripts versus 946 downregulated transcripts were annotated in these two groups ( Figure 1A,B). It was found that there are differences in tension-related genes like yes1 associated transcriptional regulator ( *YAP)*, long intergenic non-protein coding RNA ( *LINC)*, piezo type mechanosensitive ion channel component ( *PIEZO)* between the two groups ( Figure 1A,B). We used principal component analysis (PCA) and non-metric multidimensional scaling (NMDS) to analyze the differences between the two groups ( Figure 1C). GO analysis revealed that there are obvious differences in the developmental process between the two groups, while there are only minor differences in epithelial cell differentiation and epidermis cell differentiation ( Figure 1D). We also found that the cytokine-cytokine receptor interaction (KEGG analysis; Figure 1E) and signal transduction mechanisms (KOG analysis; Figure 1F) are the richest factors between the two groups. qPCR analysis showed that *LIMS2*, *PPP2R5D* and *RNF130* are significantly upregulated in HS ( Figure 1G–I). Using the small RNA sequencing, we identified a total of 2191 distinct miRNA and 120 novel miRNAs ( Supplementary Table S1). Bioinformatics analysis showed that compared with those in NTS, the expressions of 80 miRNAs, 13 piRNAs, 52 snoRNAs, 13 snRNAs, 5 repeatRNAs are significantly upregulated in HS, while the expression of 79 miRNAs, 16 piRNAs, 19 snoRNAs, 2 snRNAs, 1 repeatRNA was significantly downregulated ( Supplementary Figure S3A–E, and Supplementary Table S2). PCA analysis showed that there are differences in both number and expression level between HTS and NTS in miRNA, piRNA, snoRNA and snRNA ( Supplementary Figure S3F–J).

**Figure 1:**
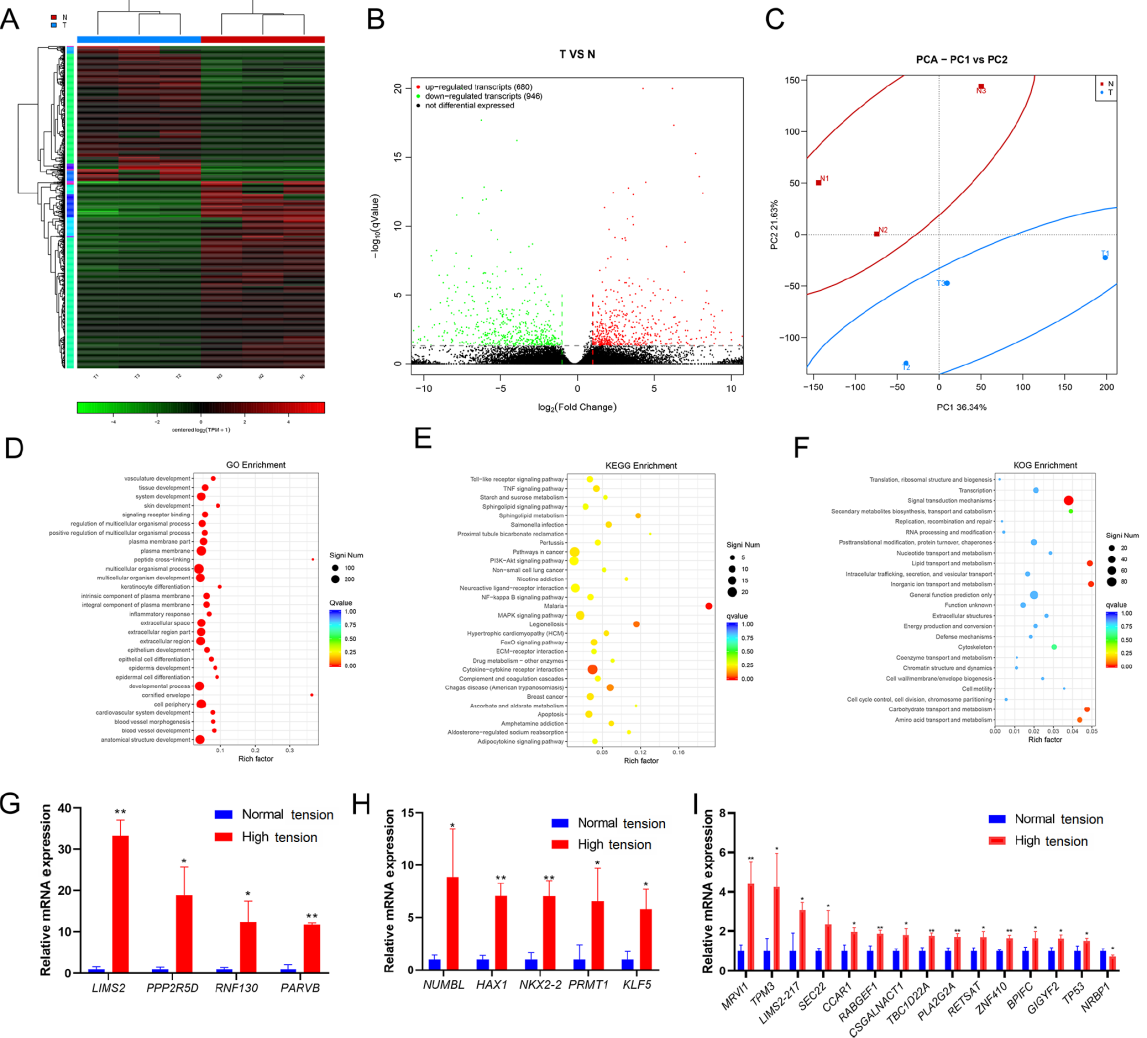
Figure 1 mRNA expression in hypertrophic scar with different tension (A) Heat map showing the differentially-expressed mRNAs in hypertrophic scar with different tension. (B) Volcanic map showing the differentially-expressed mRNAs in hypertrophic scar with different tension. (C) PCA analysis of mRNAs in hypertrophic scar with different tension. (D) GO analysis of mRNAs in hypertrophic scar with different tension. (E) KEGG analysis of mRNAs in hypertrophic scar with different tension. (F) KOG analysis of mRNAs in hypertrophic scar with different tension. (G) Upregulated mRNAs in high tension detected by qPCR. (H) Upregulated mRNAs in high tension detected by qPCR. (I) Differentially-expressed mRNAs in hypertrophic scar with different tension detected by qPCR. Data are shown as the mean±SD, * P<0.05, and ** P<0.01.

To verify these results, we performed qPCR analysis and found that two miRNAs including hsa-miR-518e-3p and hsa-novel-405 are upregulated, two miRNAs including hsa-miR-182-3p and hsa-novel-328 are downregulated ( Figure 2A), piRNA DQ587153 is upregulate and piRNA DQ570392 is downregulated ( Figure 2B), three snoRNAs including SNORD111, SNORA67 and AP000254.1 are upregulated, two snoRNAs including SNORD127 and SNORD38A are downregulated ( Figure 2C), and three snRNAs including RNU2-46P, U3, RNU6-14P are upregulated ( Figure 2D).

**Figure 2:**
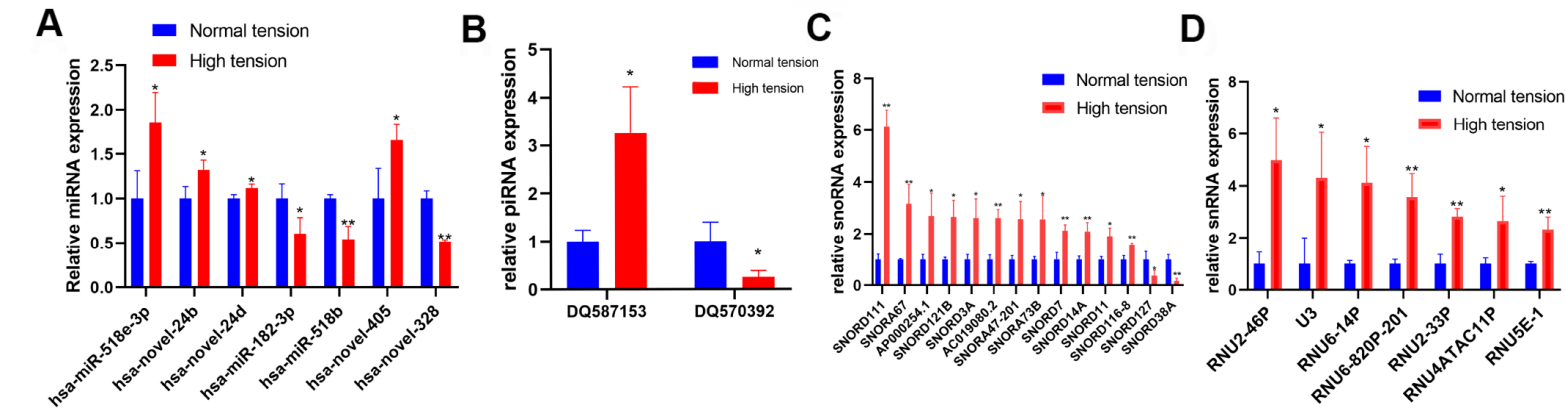
Figure 2 sncRNA expression in hypertrophic scar with different tension (A) Differentially-expressed miRNAs in hypertrophic scar with different tension detected by qPCR. (B) Differentially-expressed piRNAs in hypertrophic scar with different tension detected by qPCR. (C,D) Differentially-expressed snoRNAs and snRNAs in hypertrophic scar with different tension detected by qPCR. Data are shown as the mean±SD. * P<0.05, and ** P<0.01.

To validate the bioinformatics results, we isolated primary fibroblasts from clinically acquired human skin specimens by collagenase digestion, and incubated cells in Flexcell tension system (Flexcell International Corporation, Hillsborough, USA) to simulate the changes in tension and rhythms according to respiratory movements at the chest port. The human fibroblasts display elongated and spread morphology in the stretched group, confirming the validity of our model. Between the stretched and non-stretched group, we selected several mRNAs, miRNAs, snoRNAs, snRNAs for further *in vitro* qPCR validation. Eleven novel differently expressed mRNAs and miRNAs were validated ( Figure 3A,B) and five snoRNAs and 3 snRNAs were also validated ( Figure 3C,D).

**Figure 3:**
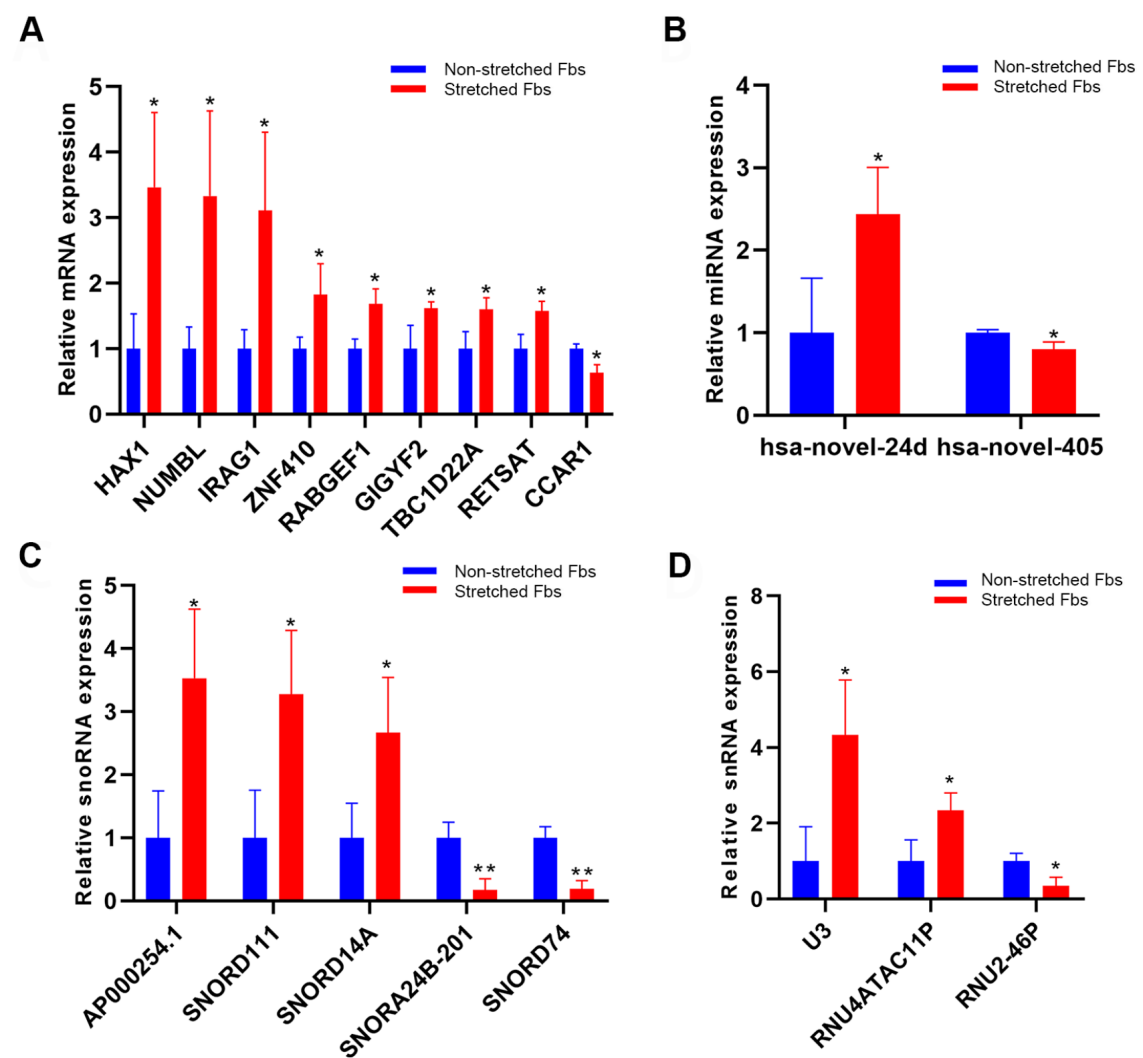
Figure 3 The mechanical sensitive gene and sncRNA expression in fibroblast with cyclic mechanical stimulation (A) The mRNA expressions in fibroblast with cyclic mechanical stimulation. (B) The miRNA expressions in fibroblast with cyclic mechanical stimulation. (C) The snoRNA expressions in fibroblast with cyclic mechanical stimulation. (D) The snRNA expressions in fibroblast with cyclic mechanical stimulation. Data are shown as the mean±SD. * P<0.05, and ** P<0.01.

In summary, we employed a tension-related hypertrophic scar specimen and performed transcriptome sequencing. Our results revealed that transcriptome differences of tension-related genes indeed exist in this specimen. We used small RNA sequencing and identified several miRNAs, piRNAs, snoRNAs, snRNAs, and repeatRNAs that are differentially expressed between HS and NTS. To validate the above results, we developed a tension model for fibroblasts and validated the sequencing results by qPCR. This is the first study to investigate the association of piRNAs, snoRNAs, snRNAs and repeatRNAs with hypertrophic scar formation, providing novel insights into the regulatory mechanism of small non-coding RNAs in the formation of abnormal scars.

## Supplementary Data

Supplementary data is available at *Acta Biochimica et Biophysica Sinica* online.

## Supporting information

Supplementary_Table_2

Supplementary_Table_1

090_supplementary_figures
