# Supplementary material for: sncRNA changes induced by tension in hypertrophic scar: sncRNAs in hypertrophic scar
Source: Acta Biochim Biophys Sin (Shanghai). 2022 Aug 5;54(8):1197–200. doi: 10.3724/abbs.2022103 (PMC9827810; doi:10.3724/abbs.2022103)
Supplement: Supplementary_Table_2 [file Supplementary_Table_2.pdf]

snRNA_ID	MeanCPM	MeanCPM	log2(T/N)	log2FoldCl	pvalue	qvalue	result
ENSG0000	4348.667	8104.667	-0.89818	-0.75163	0.047765	0.171955	down
ENSG0000	7941.333	4075.333	0.962463	1.179684	0.016268	0.089179	up
ENSG0000	1328.667	3666.333	-1.46436	-1.29872	0.018722	0.091909	down
ENSG0000	18921.33	6365.333	1.571706	1.786398	0.0003	0.002697	up
ENSG0000	84781	21220.67	1.998271	2.224682	1.02E-06	1.84E-05	up
ENSG0000	46262.67	26709	0.792522	1.008772	0.006524	0.044037	up
ENSG0000	42346.67	1807	4.55058	4.785616	2.25E-14	1.21E-12	up
ENSG0000	20092.33	8577.667	1.227988	1.443328	0.000289	0.002697	up
ENSG0000	11709.67	1122	3.383555	3.625956	2.68E-09	7.24E-08	up
ENSG0000	6324	1646.667	1.941289	2.188977	0.000843	0.0065	up
ENSG0000	2757	355.6667	2.954502	3.194123	2.93E-05	0.000396	up
ENSG0000	396	1237	-1.64327	-1.45915	0.029147	1	down
ENSG0000	2667.333	1479	0.850776	1.049176	0.042879	0.165391	up
ENSG0000	1022.667	2447	-1.25868	-1.09291	0.031511	0.134147	down
ENSG0000	6573.333	3353.667	0.970886	1.180772	0.016515	0.089179	up
