# Supplementary material for: sncRNA changes induced by tension in hypertrophic scar: sncRNAs in hypertrophic scar
Source: Acta Biochim Biophys Sin (Shanghai). 2022 Aug 5;54(8):1197–200. doi: 10.3724/abbs.2022103 (PMC9827810; doi:10.3724/abbs.2022103)
Supplement: Supplementary_Table_1 [file Supplementary_Table_1.pdf]

>hsa-let-7a-5p	UGAGGUAGUAGGUUGUAUAGUU
>hsa-let-7a-3p	CUAUACAAUCUACUGUCUUUC
>hsa-let-7a-2-3p	CUGUACAGCCUCCUAGCUUUCC
>hsa-let-7b-5p	UGAGGUAGUAGGUUGUGUGGUU
>hsa-let-7b-3p	CUAUACAACCUACUGCCUUCCC
>hsa-let-7c-5p	UGAGGUAGUAGGUUGUAUGGUU
>hsa-let-7c-3p	CUGUACAACCUUCUAGCUUUCC
>hsa-let-7d-5p	AGAGGUAGUAGGUUGCAUAGUU
>hsa-let-7d-3p	CUAUACGACCUGCUGCCUUUCU
>hsa-let-7e-5p	UGAGGUAGGAGGUUGUAUAGUU
>hsa-let-7e-3p	CUAUACGGCCUCCUAGCUUUCC
>hsa-let-7f-5p	UGAGGUAGUAGAUUGUAUAGUU
>hsa-let-7f-1-3p	CUAUACAAUCUAUUGCCUUCCC
>hsa-let-7f-2-3p	CUAUACAGUCUACUGUCUUUCC
>hsa-miR-15a-5p	UAGCAGCACAUAAUGGUUUGUG
>hsa-miR-15a-3p	CAGGCCAUAUUGUGCUGCCUCA
>hsa-miR-16-5p	UAGCAGCACGUAAAUAUUGGCG
>hsa-miR-16-1-3p	CCAGUAUUAACUGUGCUGCUGA
>hsa-miR-17-5p	CAAAGUGCUUACAGUGCAGGUAG
>hsa-miR-17-3p	ACUGCAGUGAAGGCACUUGUAG
>hsa-miR-18a-5p	UAAGGUGCAUCUAGUGCAGAUAG
>hsa-miR-18a-3p	ACUGCCCUAAGUGCUCCUUCUGG
>hsa-miR-19a-5p	AGUUUUGCAUAGUUGCACUACA
>hsa-miR-19a-3p	UGUGCAAAUCUAUGCAAAACUGA
>hsa-miR-19b-1-5p	AGUUUUGCAGGUUUGCAUCCAGC
>hsa-miR-19b-3p	UGUGCAAAUCCAUGCAAAACUGA
>hsa-miR-20a-5p	UAAAGUGCUUAUAGUGCAGGUAG
>hsa-miR-20a-3p	ACUGCAUUAUGAGCACUUAAAG
>hsa-miR-21-5p	UAGCUUAUCAGACUGAUGUUGA
>hsa-miR-21-3p	CAACACCAGUCGAUGGGCUGU
>hsa-miR-22-5p	AGUUCUUCAGUGGCAAGCUUUA
>hsa-miR-22-3p	AAGCUGCCAGUUGAAGAACUGU
>hsa-miR-23a-5p	GGGGUUCCUGGGGAUGGGAUUU
>hsa-miR-23a-3p	AUCACAUUGCCAGGGAUUUCC
>hsa-miR-24-1-5p	UGCCUACUGAGCUGAUAUCAGU
>hsa-miR-24-3p	UGGCUCAGUUCAGCAGGAACAG
>hsa-miR-24-2-5p	UGCCUACUGAGCUGAAACACAG
>hsa-miR-25-5p	AGGCGGAGACUUGGGCAAUUG
>hsa-miR-25-3p	CAUUGCACUUGUCUCGGUCUGA
>hsa-miR-26a-5p	UUCAAGUAAUCCAGGAUAGGCU
>hsa-miR-26a-1-3p	CCUAUUCUUGGUUACUUGCACG
>hsa-miR-26b-5p	UUCAAGUAAUUCAGGAUAGGU
>hsa-miR-26b-3p	CCUGUUCUCCAUUACUUGGCUC
>hsa-miR-27a-5p	AGGGCUUAGCUGCUUGUGAGCA
>hsa-miR-27a-3p	UUCACAGUGGCUAAGUUCCGC
>hsa-miR-28-5p	AAGGAGCUCACAGUCUAUUGAG
>hsa-miR-28-3p	CACUAGAUUGUGAGCUCCUGGA
>hsa-miR-29a-5p	ACUGAUUUCUUUUGGUGUUCAG
>hsa-miR-29a-3p	UAGCACCAUCUGAAAUCGGUUA
>hsa-miR-30a-5p	UGUAAACAUCCUCGACUGGAAG
>hsa-miR-30a-3p	CUUUCAGUCGGAUGUUUGCAGC
>hsa-miR-31-5p	AGGCAAGAUGCUGGCAUAGCU
>hsa-miR-31-3p	UGCUAUGCCAACAUAUUGCCAU
>hsa-miR-32-5p	UAUUGCACAUUACUAAGUUGCA
>hsa-miR-32-3p	CAAUUUAGUGUGUGUGAUAUUU
>hsa-miR-33a-5p	GUGCAUUGUAGUUGCAUUGCA
>hsa-miR-33a-3p	CAAUGUUUCCACAGUGCAUCAC
>hsa-miR-92a-1-5p	AGGUUGGGAUCGGUUGCAAUGCU

>hsa-miR-92a-3p	UAUUGCACUUGUCCCGGCCUGU
>hsa-miR-93-5p	CAAAGUGCUGUUCGUGCAGGUAG
>hsa-miR-93-3p	ACUGCUGAGCUAGCACUUCCCG
>hsa-miR-95-5p	UCAAUAAAUGUCUGUUGAAUU
>hsa-miR-95-3p	UUCAACGGGUAUUUAUUGAGCA
>hsa-miR-96-5p	UUUGGCACUAGCACAUUUUUGCU
>hsa-miR-96-3p	AAUCAUGUGCAGUGCCAAUAUG
>hsa-miR-98-5p	UGAGGUAGUAAGUUGUAUUGUU
>hsa-miR-98-3p	CUAUACAACUUACUACUUUCCC
>hsa-miR-99a-5p	AACCCGUAGAUCCGAUCUUGUG
>hsa-miR-99a-3p	CAAGCUCGCUUCUAUGGGUCUG
>hsa-miR-100-5p	AACCCGUAGAUCCGAACUUGUG
>hsa-miR-100-3p	CAAGCUUGUAUCUAUAGGUAUG
>hsa-miR-101-5p	CAGUUAUCACAGUGCUGAUGCU
>hsa-miR-101-3p	UACAGUACUGUGAUAACUGAA
>hsa-miR-29b-1-5p	GCUGGUUUCAUAUGGUGGUUUAGA
>hsa-miR-29b-3p	UAGCACCAUUUGAAAUCAGUGUU
>hsa-miR-29b-2-5p	CUGGUUUCACAUGGUGGCUUAG
>hsa-miR-103a-2-5p	AGCUUCUUUACAGUGCUGCCUUG
>hsa-miR-103a-3p	AGCAGCAUUGUACAGGGCUAUGA
>hsa-miR-105-5p	UCAAAUGCUCAGACUCCUGUGGU
>hsa-miR-106a-5p	AAAAGUGCUUACAGUGCAGGUAG
>hsa-miR-106a-3p	CUGCAAUGUAAGCACUUCUUAC
>hsa-miR-107	AGCAGCAUUGUACAGGGCUAUCA
>hsa-miR-16-2-3p	CCAAUAUUACUGUGCUGCUUUA
>hsa-miR-192-5p	CUGACCUAUGAAUUGACAGCC
>hsa-miR-192-3p	CUGCCAAUUCCAUAGGUCACAG
>hsa-miR-196a-5p	UAGGUAGUUUCAUGUUGUUGGG
>hsa-miR-197-3p	UUCACCACCUUCUCCACCCAGC
>hsa-miR-199a-5p	CCCAGUGUUCAGACUACCUGUUC
>hsa-miR-199a-3p	ACAGUAGUCUGCACAUUGGUUA
>hsa-miR-129-5p	CUUUUUGCGGUCUGGGCUUGC
>hsa-miR-129-1-3p	AAGCCCUUACCCCAAAAAGUAU
>hsa-miR-148a-5p	AAAGUUCUGAGACACUCCGACU
>hsa-miR-148a-3p	UCAGUGCACUACAGAACUUUGU
>hsa-miR-30c-5p	UGUAAACAUCCUACACUCUCAGC
>hsa-miR-30c-2-3p	CUGGGAGAAGGCUGUUUACUCU
>hsa-miR-30d-5p	UGUAAACAUCCCCGACUGGAAG
>hsa-miR-30d-3p	CUUUCAGUCAGAUGUUUGCUGC
>hsa-miR-139-5p	UCUACAGUGCACGUGUCUCCAGU
>hsa-miR-139-3p	UGGAGACGCGGCCCUGUUGGAGU
>hsa-miR-7-5p	UGGAAGACUAGUGAUUUUGUUGU
>hsa-miR-7-1-3p	CAACAAAUCACAGUCUGCCAUA
>hsa-miR-7-2-3p	CAACAAAUCCCAGUCUACCUAA
>hsa-miR-10a-5p	UACCCUGUAGAUCCGAAUUUGUG
>hsa-miR-10a-3p	CAAAUUCGUAUCUAGGGGAAUA
>hsa-miR-10b-5p	UACCCUGUAGAACCGAAUUUGUG
>hsa-miR-10b-3p	ACAGAUUCGAUUCUAGGGGAAU
>hsa-miR-34a-5p	UGGCAGUGUCUUAGCUGGUUGU
>hsa-miR-34a-3p	CAAUCAGCAAGUAUACUGCCCU
>hsa-miR-181a-5p	AACAUUCAACGCUGUCGGUGAGU
>hsa-miR-181a-2-3p	ACCACUGACCGUUGACUGUACC
>hsa-miR-181b-5p	AACAUUCAUUGCUGUCGGUGGGU
>hsa-miR-181b-3p	CUCACUGAACAAUGAAUGCAA
>hsa-miR-181c-5p	AACAUUCAACCUGUCGGUGAGU
>hsa-miR-181c-3p	AACCAUCGACCGUUGAGUGGAC
>hsa-miR-182-5p	UUUGGCAAUGGUAGAACUCACACU
>hsa-miR-182-3p	UGGUUCUAGACUUGCCAACUA

>hsa-miR-183-5p	UAUGGCACUGGUAGAAUUCACU
>hsa-miR-183-3p	GUGAAUUACCGAAGGGCCAUAA
>hsa-miR-187-5p	GGCUACAACACAGGACCCGGGC
>hsa-miR-187-3p	UCGUGUCUUGUGUUGCAGCCGG
>hsa-miR-196a-3p	CGGCAACAAGAAACUGCCUGAG
>hsa-miR-199b-5p	CCCAGUGUUUAGACUAUCUGUUC
>hsa-miR-199b-3p	ACAGUAGUCUGCACAUUGGUUA
>hsa-miR-203a-5p	AGUGGUUCUUAACAGUUCAACAGUU
>hsa-miR-203a-3p	GUGAAAUGUUUAGGACCACUAG
>hsa-miR-204-5p	UUCCCUUUGUCAUCCUAUGCCU
>hsa-miR-204-3p	GCUGGGAAGGCAAAGGGACGU
>hsa-miR-205-5p	UCCUUCAUUCCACCGGAGUCUG
>hsa-miR-205-3p	GAUUUCAGUGGAGUGAAGUUC
>hsa-miR-210-5p	AGCCCCUGCCCACCGCACACUG
>hsa-miR-210-3p	CUGUGCGUGUGACAGCGGCUGA
>hsa-miR-211-5p	UUCCCUUUGUCAUCCUUCGCCU
>hsa-miR-211-3p	GCAGGGACAGCAAAGGGGUGC
>hsa-miR-212-5p	ACCUUGGCUCUAGACUGCUUACU
>hsa-miR-212-3p	UAACAGUCUCCAGUCACGGCC
>hsa-miR-181a-3p	ACCAUCGACCGUUGAUUGUACC
>hsa-miR-214-5p	UGCCUGUCUACACUUGCUGUGC
>hsa-miR-214-3p	ACAGCAGGCACAGACAGGCAGU
>hsa-miR-215-5p	AUGACCUAUGAAUUGACAGAC
>hsa-miR-216a-5p	UAAUCUCAGCUGGCAACUGUGA
>hsa-miR-216a-3p	UCACAGUGGUCUCUGGGAUUAU
>hsa-miR-217	UACUGCAUCAGGAACUGAUUGGA
>hsa-miR-218-5p	UUGUGCUUGAUCUAACCAUGU
>hsa-miR-218-1-3p	AUGGUUCCGUCAAGCACCAUGG
>hsa-miR-218-2-3p	CAUGGUUCUGUCAAGCACCGCG
>hsa-miR-219a-5p	UGAUUGUCCAAACGCAAUUCU
>hsa-miR-219a-1-3p	AGAGUUGAGUCUGGACGUCCCG
>hsa-miR-221-5p	ACCUGGCAUACAAUGUAGAUUU
>hsa-miR-221-3p	AGCUACAUUGUCUGCUGGGUUUC
>hsa-miR-222-5p	CUCAGUAGCCAGUGUAGAUCCU
>hsa-miR-222-3p	AGCUACAUCUGGCUACUGGGU
>hsa-miR-223-5p	CGUGUAUUUGACAAGCUGAGUU
>hsa-miR-223-3p	UGUCAGUUUGUCAAAUACCCCA
>hsa-miR-224-5p	CAAGUCACUAGUGGUUCCGUU
>hsa-miR-224-3p	AAAAUGGUGCCCUAGUGACUACA
>hsa-miR-200b-5p	CAUCUUACUGGGCAGCAUUGGA
>hsa-miR-200b-3p	UAAUACUGCCUGGUAAUGAUGA
>hsa-let-7g-5p	UGAGGUAGUAGUUUGUACAGUU
>hsa-let-7g-3p	CUGUACAGGCCACUGCCUUGC
>hsa-let-7i-5p	UGAGGUAGUAGUUUGUGCUGUU
>hsa-let-7i-3p	CUGCGCAAGCUACUGCCUUGCU
>hsa-miR-1-3p	UGGAAUGUAAAGAAGUAUGUAU
>hsa-miR-15b-5p	UAGCAGCACAUCAUGGUUUACA
>hsa-miR-15b-3p	CGAAUCAUUAUUUGCUGCUCUA
>hsa-miR-23b-5p	UGGGUUCCUGGCAUGCUGAUUU
>hsa-miR-23b-3p	AUCACAUUGCCAGGGAUUACC
>hsa-miR-27b-5p	AGAGCUUAGCUGAUUGGUGAAC
>hsa-miR-27b-3p	UUCACAGUGGCUAAGUUCUGC
>hsa-miR-30b-5p	UGUAAACAUCCUACACUCAGCU
>hsa-miR-30b-3p	CUGGGAGGUGGAUGUUUACUUC
>hsa-miR-122-5p	UGGAGUGUGACAAUGGUGUUUG
>hsa-miR-122-3p	AACGCCAUUAUCACACUAAAUA
>hsa-miR-124-3p	UAAGGCACGCGGUGAAUGCC
>hsa-miR-125b-5p	UCCCUGAGACCCUAACUUGUGA

>hsa-miR-125b-1-3p ACGGGUUAGGCUCUUGGGAGCU
>hsa-miR-128-1-5p CGGGGCCGUAGCACUGUCUGAGA
>hsa-miR-128-3p UCACAGUGAACCGGUCUCUUU
>hsa-miR-130a-3p CAGUGCAAUGUUAAAAGGGCAU
>hsa-miR-132-5p ACCGUGGCUUUCGAUUGUUACU
>hsa-miR-132-3p UAACAGUCUACAGCCAUGGUCG
>hsa-miR-133a-5p AGCUGGUAAAAUGGAACCAAAU
>hsa-miR-133a-3p UUUGGUCCCCUUCAACCAGCUG
>hsa-miR-135a-5p UAUGGCUUUUUAUUCCUAUGUGA
>hsa-miR-137 UUAUUGCUUAAGAAUACGCGUAG
>hsa-miR-138-5p AGCUGGUGUUGUGAAUCAGGCCG
>hsa-miR-140-5p CAGUGGUUUUACCCUAUGGUAG
>hsa-miR-140-3p UACCACAGGGUAGAACCACGG
>hsa-miR-141-5p CAUCUUCCAGUACAGUGUUGGA
>hsa-miR-141-3p UAACACUGUCUGGUAAAGAUGG
>hsa-miR-142-5p CAUAAAGUAGAAAGCACUACU
>hsa-miR-142-3p UGUAGUGUUUCCUACUUUAUGGA
>hsa-miR-143-5p GGUGCAGUGCUGCAUCUCUGGU
>hsa-miR-143-3p UGAGAUGAAGCACUGUAGCUC
>hsa-miR-144-5p GGAUAUCAUCAUAUACUGUAAG
>hsa-miR-144-3p UACAGUAUAGAUGAUGUACU
>hsa-miR-145-5p GUCCAGUUUUCCCAGGAAUCCCU
>hsa-miR-145-3p GGAUUCCUGGAAAUACUGUUCU
>hsa-miR-152-5p AGGUUCUGUGAUACACUCCGACU
>hsa-miR-152-3p UCAGUGCAUGACAGAACUUGG
>hsa-miR-153-3p UUGCAUAGUCACAAAAGUGAUC
>hsa-miR-153-5p UCAUUUUUGUGAUGUUGCAGCU
>hsa-miR-191-5p CAACGGAAUCCCAAAAGCAGCUG
>hsa-miR-191-3p GCUGCGCUUGGAUUUCGUCCCC
>hsa-miR-9-5p UCUUUGGUUAUCUAGCUGUAUGA
>hsa-miR-9-3p AUAAAGCUAGAUAACCGAAAGU
>hsa-miR-125a-5p UCCCUGAGACCCUUUAACCUGUGA
>hsa-miR-125a-3p ACAGGUGAGGUUCUUGGGAGCC
>hsa-miR-125b-2-3p UCACAAGUCAGGCUCUUGGGAC
>hsa-miR-126-5p CAUUAUUACUUUUGGUACGCG
>hsa-miR-126-3p UCGUACCGUGAGUAAUAAUGCG
>hsa-miR-127-5p CUGAAGCUCAGAGGGCUCUGAU
>hsa-miR-127-3p UCGGAUCCGUCUGAGCUUGGCU
>hsa-miR-129-2-3p AAGCCCUUACCCCAAAAAGCAU
>hsa-miR-134-5p UGUGACUGGUUGACCAGAGGGG
>hsa-miR-134-3p CCUGUGGGCCACCUAGUCACCAA
>hsa-miR-136-5p ACUCCAUUUGUUUUGAUGAUGGA
>hsa-miR-136-3p CAUCAUCGUCUCAAAUGAGUCU
>hsa-miR-146a-5p UGAGAACUGAAUUCCAUGGGUU
>hsa-miR-146a-3p CCUCUGAAAUUCAGUUCUUCAG
>hsa-miR-149-5p UCUGGCUCCGUGUCUUCACUCCC
>hsa-miR-150-5p UCUCCCAACCCUUGUACCAGUG
>hsa-miR-150-3p CUGGUACAGGCCUGGGGGACAG
>hsa-miR-154-5p UAGGUUAUCCGUGUUGCCUUCG
>hsa-miR-154-3p AAUCAUACACGGUUGACCUAUU
>hsa-miR-184 UGGACGGAGAACUGAUAAGGGU
>hsa-miR-185-5p UGGAGAGAAAGGCAGUUCCUGA
>hsa-miR-185-3p AGGGGCUGGCUUUCCUCUGGUC
>hsa-miR-186-5p CAAAGAAUUCUCCUUUUGGGCU
>hsa-miR-186-3p GCCCAAAGGUGAAUUUUUUGGG
>hsa-miR-188-5p CAUCCCUUGCAUGGUGGAGGG
>hsa-miR-188-3p CUCCCACAUGCAGGGUUUGCA
>hsa-miR-190a-5p UGAUAUGUUUGAUAUAUUAGGU

>hsa-miR-190a-3p	CUAUAUAUCAAACAUAUUCCU
>hsa-miR-193a-5p	UGGGUCUUUGCGGGCGAGAUGA
>hsa-miR-193a-3p	AACUGGCCUACAAAGUCCCAGU
>hsa-miR-194-5p	UGUAACAGCAACUCCAUGUGGA
>hsa-miR-195-5p	UAGCAGCACAGAAAUAUUGGC
>hsa-miR-195-3p	CCAAUAUUGGCUGUGCUGCUCC
>hsa-miR-206	UGGAAUGUAAGGAAGUGUGUGG
>hsa-miR-320a	AAAAGCUGGGUUGAGAGGGCGA
>hsa-miR-200c-5p	CGUCUUACCCAGCAGUGUUUGG
>hsa-miR-200c-3p	UAAUACUGCCGGGUAAUGAUGGA
>hsa-miR-155-5p	UUAAUGCUAAUCGUGAUAGGGGU
>hsa-miR-155-3p	CUCCUACAUAUUAGCAUUAACA
>hsa-miR-181b-2-3p	CUCACUGAUCAAUGAAUGCA
>hsa-miR-194-3p	CCAGUGGGGCUGCUGUUAUCUG
>hsa-miR-106b-5p	UAAAGUGCUGACAGUGCAGAU
>hsa-miR-106b-3p	CCGCACUGUGGGUACUUGCUGC
>hsa-miR-29c-5p	UGACCGAUUUCUCCUGGUGUUC
>hsa-miR-29c-3p	UAGCACCAUUUGAAAUCGGUUA
>hsa-miR-30c-1-3p	CUGGGAGAGGGUUGUUUACUCC
>hsa-miR-200a-5p	CAUCUUACCGGACAGUGCUGGA
>hsa-miR-200a-3p	UAACACUGUCUGGUAACGAUGU
>hsa-miR-34b-5p	UAGGCAGUGUCAUUAGCUGAUUG
>hsa-miR-34b-3p	CAAUCACUAACUCCACUGCCAU
>hsa-miR-34c-5p	AGGCAGUGUAGUUAGCUGAUUGC
>hsa-miR-34c-3p	AAUCACUAACCACACGGCCAGG
>hsa-miR-299-5p	UGGUUUACCGUCCCACAUACAU
>hsa-miR-299-3p	UAUGUGGGAUGGUAAACCGCUU
>hsa-miR-301a-5p	GCUCUGACUUUAUUGCACUACU
>hsa-miR-301a-3p	CAGUGCAAUAGUAUUGUCAAAGC
>hsa-miR-99b-5p	CACCCGUAGAACCGACCUUGCG
>hsa-miR-99b-3p	CAAGCUCGUGUCUGUGGGUCCG
>hsa-miR-296-5p	AGGGCCCCCCCUCAAUCCUGU
>hsa-miR-296-3p	GAGGGUUGGGUGGAGGCUCUCC
>hsa-miR-130b-5p	ACUCUUUCCCUGUUGCACUAC
>hsa-miR-130b-3p	CAGUGCAAUGAUGAAAGGGCAU
>hsa-miR-30e-5p	UGUAAACAUCCUUGACUGGAAG
>hsa-miR-30e-3p	CUUUCAGUCGGAUGUUUACAGC
>hsa-miR-26a-2-3p	CCUAUUCUUGAUUACUUGUUUC
>hsa-miR-361-5p	UUAUCAGAAUCUCCAGGGGUAC
>hsa-miR-361-3p	UCCCCCAGGUGUGAUUCUGAUUU
>hsa-miR-362-5p	AAUCCUUGGAACCUAGGUGUGAGU
>hsa-miR-362-3p	AACACACCUAUUCAAGGAUUCA
>hsa-miR-363-5p	CGGGUGGAUCACGAUGCAAUUU
>hsa-miR-363-3p	AAUUGCACGGUAUCCAUCUGUA
>hsa-miR-365a-5p	AGGGACUUUUGGGGGCAGAUGUG
>hsa-miR-365a-3p	UAAUGCCCCUAAAAAUCCUUAU
>hsa-miR-365b-5p	AGGGACUUUCAGGGGCAGCUGU
>hsa-miR-365b-3p	UAAUGCCCCUAAAAAUCCUUAU
>hsa-miR-376c-5p	GGUGGAUAUUCCUUCUAUGUU
>hsa-miR-376c-3p	AACAUAGAGGAAAUUCCACGU
>hsa-miR-369-5p	AGAUCGACCGUGUUAUAUUCGC
>hsa-miR-369-3p	AAUAAUACAUGGUUGAUCUUU
>hsa-miR-370-5p	CAGGUCACGUCUCUGCAGUUAC
>hsa-miR-370-3p	GCCUGCUGGGGUGGAACCUGGU
>hsa-miR-371a-5p	ACUCAAACUGUGGGGGCACU
>hsa-miR-372-3p	AAAGUGCUGCGACAUUUGAGCGU
>hsa-miR-374a-5p	UUAUAAUACAACCUGAUAAGUG
>hsa-miR-374a-3p	CUUAUCAGAUUGUAUUGUAAUU

>hsa-miR-375	UUUGUUCGUUCGGCUCGCGUGA
>hsa-miR-376a-5p	GUAGAUUCUCCUUCUAUGAGUA
>hsa-miR-376a-3p	AUCAUAGAGGAAAAUCCACGU
>hsa-miR-377-5p	AGAGGUUGCCCUUGGUGAAUUC
>hsa-miR-377-3p	AUCACACAAAGGCAACUUUUGU
>hsa-miR-378a-5p	CUCCUGACUCCAGGUCCUGUGU
>hsa-miR-378a-3p	ACUGGACUUGGAGUCAGAAGGC
>hsa-miR-379-5p	UGGUAGACUAUGGAACGUAGG
>hsa-miR-379-3p	UAUGUAACAUGGUCCACUAACU
>hsa-miR-380-5p	UGGUUGACCAUAGAACAUGCGC
>hsa-miR-380-3p	UAUGUAAUAUGGUCCACAUCUU
>hsa-miR-381-5p	AGCGAGGUUGCCCUUUGUAUAU
>hsa-miR-381-3p	UAUACAAGGGCAAGCUCUCUGU
>hsa-miR-382-5p	GAAGUUGUUCGUGGUGGAUUCG
>hsa-miR-382-3p	AAUCAUUCACGGACAACACUU
>hsa-miR-383-5p	AGAUCAGAAGGUGAUUGUGGCU
>hsa-miR-383-3p	ACAGCACUGCCUGGUCAGA
>hsa-miR-340-5p	UUAUAAAGCAAUGAGACUGAUU
>hsa-miR-340-3p	UCCGUCUCAGUUACUUUAUAGC
>hsa-miR-330-5p	UCUCUGGGCCUGUGUCUUAGGC
>hsa-miR-330-3p	GCAAAGCACACGGCCUGCAGAGA
>hsa-miR-328-3p	CUGGCCCUCUCUGCCCUUCCGU
>hsa-miR-342-5p	AGGGGUGCUAUCUGUGAUUGA
>hsa-miR-342-3p	UCUCACACAGAAAUCGCACCCGU
>hsa-miR-337-5p	GAACGGCUUCAUACAGGAGUU
>hsa-miR-337-3p	CUCCUAUAUGAUGCCUUUCUUC
>hsa-miR-323a-5p	AGGUGGUCCGUGGCGCGUUCGC
>hsa-miR-323a-3p	CACAUUACACGGUCGACCUCU
>hsa-miR-326	CCUCUGGGCCCUUCCUCCAG
>hsa-miR-151a-5p	UCGAGGAGCUCACAGUCUAGU
>hsa-miR-151a-3p	CUAGACUGAAGCUCCUUGAGG
>hsa-miR-135b-5p	UAUGGCUUUUCAUUCCUAUGUGA
>hsa-miR-135b-3p	AUGUAGGGCUAAAAGCCAUGGG
>hsa-miR-148b-5p	AAGUUCUGUUAUACACUCAGGC
>hsa-miR-148b-3p	UCAGUGCAUCACAGAACUUUGU
>hsa-miR-331-5p	CUAGGUAUGGUCCCAGGGAUCC
>hsa-miR-331-3p	GCCCCUGGGCCUAUCCUAGAA
>hsa-miR-324-5p	CGCAUCCCCUAGGGCAUUGGUGU
>hsa-miR-324-3p	ACUGCCCCAGGUGCUGCUGG
>hsa-miR-338-5p	AACAAUAUCCUGGUGCUGAGUG
>hsa-miR-338-3p	UCCAGCAUCAGUGAUUUUGUUG
>hsa-miR-339-5p	UCCCUGUCCUCCAGGAGCUCACG
>hsa-miR-339-3p	UGAGCGCCUCGACGACAGAGCCG
>hsa-miR-335-5p	UCAAGAGCAAUAACGAAAAAUGU
>hsa-miR-335-3p	UUUUUCAUUAUUGCUCCUGACC
>hsa-miR-133b	UUUGGUCCCCUUCAACCAGCUA
>hsa-miR-345-5p	GCUGACUCCUAGUCCAGGGCUC
>hsa-miR-346	UGUCUGCCCGCAUGCCUGCCUCU
>hsa-miR-196b-5p	UAGGUAGUUUCCUGUUGUUGGG
>hsa-miR-196b-3p	UCGACAGCACGACACUGCCUUC
>hsa-miR-422a	ACUGGACUUAGGGUCAGAAGGC
>hsa-miR-423-5p	UGAGGGGCAGAGAGCGAGACUUU
>hsa-miR-423-3p	AGCUCGGUCUGAGGCCCCUCAGU
>hsa-miR-424-5p	CAGCAGCAAUUCAUGUUUUGAA
>hsa-miR-424-3p	CAAAACGUGAGGCGCUGCUAU
>hsa-miR-425-5p	AAUGACACGAUCACUCCCGUUGA
>hsa-miR-425-3p	AUCGGGAAUGUCGUGUCCGCCC
>hsa-miR-18b-5p	UAAGGUGCAUCUAGUGCAGUUAG

>hsa-miR-18b-3p	UGCCCUAAAUGCCCCUUCUGGC
>hsa-miR-20b-5p	CAAAGUGCUCAUAGUGCAGGUAG
>hsa-miR-20b-3p	ACUGUAGUAUGGGCACUUCCAG
>hsa-miR-429	UAAUACUGUCUGGUAAAACCGU
>hsa-miR-449a	UGGCAGUGUAUUGUUAGCUGGU
>hsa-miR-450a-5p	UUUUGCGAUGUGUUCCUAAUAU
>hsa-miR-450a-1-3p	AUUGGGAACAUUUUGCAUGUAU
>hsa-miR-431-5p	UGUCUUGCAGGCCGUCAUGCA
>hsa-miR-431-3p	CAGGUCGUCUUGCAGGGCUUCU
>hsa-miR-433-5p	UACGGUGAGCCUGUCAUUAUUC
>hsa-miR-433-3p	AUCAUGAUGGGCUCCUCGGUGU
>hsa-miR-329-5p	GAGGUUUUCUGGGUUUCUGUUUC
>hsa-miR-329-3p	AACACACCUGGUUAACCUCUUU
>hsa-miR-451a	AAACCGUUACCAUUACUGAGUU
>hsa-miR-452-5p	AACUGUUUGCAGAGGAAACUGA
>hsa-miR-409-5p	AGGUUACCCGAGCAACUUUGCAU
>hsa-miR-409-3p	GAAUGUUGCUCGGUGAACCCCU
>hsa-miR-412-5p	UGGUCGACCAGUUGGAAAGUAAU
>hsa-miR-412-3p	ACUUCACCUGGUCCACUAGCCGU
>hsa-miR-410-5p	AGGUUGUCUGUGAUGAGUUCG
>hsa-miR-410-3p	AAUAUAACACAGAUGGCCUGU
>hsa-miR-376b-5p	CGUGGAUAUUCCUUCUAUGUUU
>hsa-miR-376b-3p	AUCAUAGAGGAAAAUCCAUGUU
>hsa-miR-483-5p	AAGACGGGAGGAAAGAAGGGAG
>hsa-miR-483-3p	UCACUCCUCUCCUCCCGUCUU
>hsa-miR-484	UCAGGCUCAGUCCCCUCCCGAU
>hsa-miR-485-5p	AGAGGCUGGCCGUGAUGAAUUC
>hsa-miR-485-3p	GUCAUACACGGCUCUCCUCUCU
>hsa-miR-486-5p	UCCUGUACUGAGCUGCCCCGAG
>hsa-miR-486-3p	CGGGGCAGCUCAGUACAGGAU
>hsa-miR-487a-5p	GUGGUUAUCCCUGCUGUGUUCG
>hsa-miR-487a-3p	AAUCAUACAGGGACAUCCAGUU
>hsa-miR-488-5p	CCCAGAUAAUGGCACUCUCAA
>hsa-miR-488-3p	UUGAAAGGCUAUUUCUUGGUC
>hsa-miR-489-5p	GGUCGUAUGUGUGACGCCAUUU
>hsa-miR-489-3p	GUGACAUCACAUAUACGGCAGC
>hsa-miR-490-3p	CAACCUGGAGGACUCCAUGCUG
>hsa-miR-491-5p	AGUGGGGAACCCUUCCAUGAGG
>hsa-miR-491-3p	CUUAUGCAAGAUUCCCUUCUAC
>hsa-miR-511-5p	GUGUCUUUUGCUCUGCAGUCA
>hsa-miR-511-3p	AAUGUGUAGCAAAAGACAGA
>hsa-miR-146b-5p	UGAGAACUGAAUUCCAUAGGCU
>hsa-miR-146b-3p	UGCCCUGUGGACUCAGUUCUGG
>hsa-miR-202-5p	UUCCUAUGCAUAUACUUCUUUG
>hsa-miR-202-3p	AGAGGUAUAGGGCAUGGGAA
>hsa-miR-492	AGGACCUGCGGGACAAGAUUCUU
>hsa-miR-493-5p	UUGUACAUGGUAGGCUUUCAUU
>hsa-miR-493-3p	UGAAGGUCUACUGUGUGCCAGG
>hsa-miR-432-5p	UCUUGGAGUAGGUCAUUGGGUGG
>hsa-miR-432-3p	CUGGAUGGCUCCUCCAUGUCU
>hsa-miR-494-5p	AGGUUGUCCGUGUUGUCUUCUCU
>hsa-miR-494-3p	UGAAACAUACACGGGAAACCUC
>hsa-miR-495-5p	GAAGUUGCCCAUGUUAUUUUCG
>hsa-miR-495-3p	AAACAAACAUGGUGCACUUCUU
>hsa-miR-496	UGAGUAUUACAUGGCCAAUCUC
>hsa-miR-193b-5p	CGGGGUUUUGAGGGCGAGAUGA
>hsa-miR-193b-3p	AACUGGCCCUCAAAGUCCCGCU
>hsa-miR-497-5p	CAGCAGCACACUGUGGUUUGU

>hsa-miR-497-3p	CAAACCACACUGUGGUGUUAGA
>hsa-miR-181d-5p	AACAUUCAUUGUUGUCGGUGGGU
>hsa-miR-181d-3p	CCACCGGGGGAUGAAUGUCAC
>hsa-miR-512-5p	CACUCAGCCUUGAGGGCACUUUC
>hsa-miR-512-3p	AAGUGCUGUCAUAGCUGAGGUC
>hsa-miR-498	UUUCAAGCCAGGGGGCGUUUUUC
>hsa-miR-520e	AAAGUGCUUCCUUUUUGAGGG
>hsa-miR-515-5p	UUCUCCAAAAGAAAGCACUUUCUG
>hsa-miR-515-3p	GAGUGCCUUCUUUUGGAGCGUU
>hsa-miR-519e-5p	UUCUCCAAAAGGGAGCACUUUC
>hsa-miR-520f-3p	AAGUGCUUCCUUUUAGAGGGUU
>hsa-miR-519c-5p	CUCUAGAGGGAAGCGCUUUCUG
>hsa-miR-519c-3p	AAAGUGCAUCUUUUUAGAGGAU
>hsa-miR-520a-5p	CUCCAGAGGGAAGUACUUUCU
>hsa-miR-520a-3p	AAAGUGCUUCCCUUUGGACUGU
>hsa-miR-526b-5p	CUCUUGAGGGAAGCACUUUCUGU
>hsa-miR-526b-5p	CUCUUGAGGGAAGCACUUUCUGU
>hsa-miR-526b-3p	GAAAGUGCUUCCUUUUAGAGGC
>hsa-miR-519b-5p	CUCUAGAGGGAAGCGCUUUCUG
>hsa-miR-519b-3p	AAAGUGCAUCCUUUUAGAGGUU
>hsa-miR-525-5p	CUCCAGAGGGAUGCACUUUCU
>hsa-miR-523-5p	CUCUAGAGGGAAGCGCUUUCUG
>hsa-miR-523-3p	GAACGCGCUUCCCUAUAGAGGGU
>hsa-miR-518f-5p	CUCUAGAGGGAAGCACUUUCUC
>hsa-miR-518f-3p	GAAAGCGCUUCUCUUUAGAGG
>hsa-miR-520b	AAAGUGCUUCCUUUUAGAGGG
>hsa-miR-518b	CAAAGCGCUCCCCUUUAGAGGU
>hsa-miR-526a	CUCUAGAGGGAAGCACUUUCUG
>hsa-miR-520c-5p	CUCUAGAGGGAAGCACUUUCUG
>hsa-miR-520c-3p	AAAGUGCUUCCUUUUAGAGGGU
>hsa-miR-518c-5p	UCUCUGGAGGGAAGCACUUUCUG
>hsa-miR-518c-3p	CAAAGCGCUUCUCUUUAGAGUGU
>hsa-miR-524-5p	CUACAAAGGGAAGCACUUUCUC
>hsa-miR-517-5p	CCUCUAGAUGGAAGCACUGUCU
>hsa-miR-517a-3p	AUCGUGCAUCCCUUUAGAGUGU
>hsa-miR-519d-5p	CCUCCAAAGGGAAGCGCUUUCUGUU
>hsa-miR-519d-3p	CAAAGUGCCUCCCUUUAGAGUG
>hsa-miR-521	AACGCACUUCCCUUUAGAGUGU
>hsa-miR-520d-5p	CUACAAAGGGAAGCCCUUUC
>hsa-miR-520d-3p	AAAGUGCUUCUCUUUGGUGGGU
>hsa-miR-517b-3p	AUCGUGCAUCCCUUUAGAGUGU
>hsa-miR-520g-5p	UCUAGAGGAAGCACUUUCUGUUU
>hsa-miR-520g-3p	ACAAAGUGCUUCCCUUUAGAGUGU
>hsa-miR-516b-5p	AUCUGGAGGUAAGAAGCACUUU
>hsa-miR-518e-5p	CUCUAGAGGGAAGCGCUUUCUG
>hsa-miR-518e-3p	AAAGCGCUUCCCUUCAGAGUG
>hsa-miR-518a-5p	CUGCAAAGGGAAGCCCUUUC
>hsa-miR-518a-3p	GAAAGCGCUUCCCUUUGCUGGA
>hsa-miR-518d-5p	CUCUAGAGGGAAGCACUUUCUG
>hsa-miR-517c-3p	AUCGUGCAUCCUUUUAGAGUGU
>hsa-miR-520h	ACAAAGUGCUUCCCUUUAGAGU
>hsa-miR-522-5p	CUCUAGAGGGAAGCGCUUUCUG
>hsa-miR-522-3p	AAAAUGGUUCCCUUUAGAGUGU
>hsa-miR-519a-5p	CUCUAGAGGGAAGCGCUUUCUG
>hsa-miR-519a-3p	AAAGUGCAUCCUUUUAGAGUGU
>hsa-miR-527	CUGCAAAGGGAAGCCCUUUC
>hsa-miR-516a-5p	UUCUCGAGGAAAGAAGCACUUUC
>hsa-miR-499a-5p	UUAAGACUUGCAGUGAUGUUU

>hsa-miR-499a-3p	AACAUCACAGCAAGUCUGUGCU
>hsa-miR-500a-5p	UAAUCCUUGCUACCUGGGUGAGA
>hsa-miR-500a-3p	AUGCACCUGGGCAAGGAUUCUG
>hsa-miR-501-5p	AAUCCUUUGUCCCUGGGUGAGA
>hsa-miR-501-3p	AAUGCACCCGGGCAAGGAUUCU
>hsa-miR-502-5p	AUCCUUGCUAUCUGGGUGCUA
>hsa-miR-502-3p	AAUGCACCUGGGCAAGGAUUCA
>hsa-miR-450a-2-3p	AUUGGGGACAUUUUGCAUUCAU
>hsa-miR-503-5p	UAGCAGCGGGAACAGUUCUGCAG
>hsa-miR-503-3p	GGGGUAUUGUUUCCGCUGCCAGG
>hsa-miR-504-5p	AGACCCUGGUCUGCACUCUAUC
>hsa-miR-505-5p	GGGAGCCAGGAAGUAUUGAUGU
>hsa-miR-505-3p	CGUCAACACUUGCUGGUUUCCU
>hsa-miR-513a-5p	UUCACAGGGAGGUGUCAU
>hsa-miR-513a-3p	UAAAUUUCACCUUUCUGAGAAGG
>hsa-miR-506-5p	UAUUCAGGAAGGUGUUACUUAA
>hsa-miR-506-3p	UAAGGCACCCUUCUGAGUAGA
>hsa-miR-507	UUUUGCACCUUUUGGAGUGAA
>hsa-miR-508-5p	UACUCCAGAGGGCGUCACUCAUG
>hsa-miR-508-3p	UGAUUGUAGCCUUUUGGAGUAGA
>hsa-miR-509-5p	UACUGCAGACAGUGGCAAUCA
>hsa-miR-509-3p	UGAUUGGUACGUCUGUGGGUAG
>hsa-miR-510-5p	UACUCAGGAGAGUGGCAAUCAC
>hsa-miR-510-3p	AUUGAAACCUCUAAGAGUGGA
>hsa-miR-514a-5p	UACUCUGGAGAGUGACAAUCAUG
>hsa-miR-514a-3p	AUUGACACUUCUGUGAGUAGA
>hsa-miR-532-5p	CAUGCCUUGAGUGUAGGACCGU
>hsa-miR-532-3p	CCUCCCACACCCAAGGCUUGCA
>hsa-miR-455-5p	UAUGUGCCUUUGGACUACAUCG
>hsa-miR-455-3p	GCAGUCCAUGGGCAUAUACAC
>hsa-miR-539-5p	GGAGAAAUUAUCCUUGGUGUGU
>hsa-miR-539-3p	AUCAUACAAGGACAAUUUCUUU
>hsa-miR-544a	AUUCUGCAUUUUUAGCAAGUUC
>hsa-miR-545-5p	UCAGUAAAUGUUUAUUAGAUGA
>hsa-miR-545-3p	UCAGCAAACAUUUAUUGUGUGC
>hsa-miR-376a-2-5p	GGUAGAUUUUCCUUCUAUGGU
>hsa-miR-487b-5p	GUGGUUAUCCCUGUCCUGUUCG
>hsa-miR-487b-3p	AAUCGUACAGGGUCAUCCACUU
>hsa-miR-551a	GCGACCCACUCUUGGUUUCCA
>hsa-miR-552-5p	GUUUAACCUUUUGCCUGUUGG
>hsa-miR-552-3p	AACAGGUGACUGGUUAGACAA
>hsa-miR-92b-5p	AGGGACGGGACGCGGUGCAGUG
>hsa-miR-92b-3p	UAUUGCACUCGUCCCGGCCUCC
>hsa-miR-556-5p	GAUGAGCUCAUUGUAAUAUGAG
>hsa-miR-556-3p	AUAUUACCAUUAGCUCAUCUUU
>hsa-miR-561-5p	AUCAAGGAUCUUAAACUUUGCC
>hsa-miR-561-3p	CAAAGUUUAAGAUCCUUGAAGU
>hsa-miR-566	GGGCGCCUGUGAUCCCAAC
>hsa-miR-551b-5p	GAAAUCAAGCGUGGGUGAGACC
>hsa-miR-551b-3p	GCGACCCAUACUUGGUUUCAG
>hsa-miR-570-5p	AAAGGUAAUUGCAGUUUUUCCC
>hsa-miR-570-3p	CGAAAACAGCAAUUACCUUUGC
>hsa-miR-573	CUGAAGUGAUGUGUAACUGAUCAG
>hsa-miR-574-5p	UGAGUGUGUGUGUGUGAGUGUGU
>hsa-miR-574-3p	CACGCUCAUGCACACACCCACA
>hsa-miR-576-5p	AUUCUAAUUUCUCCACGUCUUU
>hsa-miR-576-3p	AAGAUGUGGAAAAAUUGGAAUC
>hsa-miR-577	UAGAUAAAAUAUUGGUACCUG

>hsa-miR-579-5p	UCGCGGUUUGUGCCAGAUGACG
>hsa-miR-579-3p	UUCAUUUGGUAUAAACCGCGAUU
>hsa-miR-580-5p	UAAUGAUUCAUCAGACUCAGAU
>hsa-miR-580-3p	UUGAGAAUGAUGAAUCAUUAGG
>hsa-miR-581	UCUUGUGUUCUCUAGAUCAGU
>hsa-miR-582-5p	UUACAGUUGUUCAACCAGUUACU
>hsa-miR-582-3p	UAACUGGUUGAACAACUGAACC
>hsa-miR-584-5p	UUAUGGUUUGCCUGGGACUGAG
>hsa-miR-584-3p	UCAGUUCCAGGCCAACCAGGCU
>hsa-miR-585-5p	CUAGCACACAGAUACGCCCAGA
>hsa-miR-585-3p	UGGGCGUAUCUGUAUGCUA
>hsa-miR-548a-3p	CAAAACUGGCAAUUACUUUUGC
>hsa-miR-586	UAUGCAUUGUAUUUUUAGGUCC
>hsa-miR-548b-5p	AAAAGUAAUUGUGGUUUUGGCC
>hsa-miR-548b-3p	CAAGAACCUCAGUUGCUUUUGU
>hsa-miR-589-5p	UGAGAACCACGUCUGCUCUGAG
>hsa-miR-589-3p	UCAGAACAAAUGCCGGUUCCCAGA
>hsa-miR-550a-5p	AGUGCCUGAGGGAGUAAGAGCCC
>hsa-miR-550a-3p	UGUCUUACUCCCUCAGGCACAU
>hsa-miR-590-5p	GAGCUUAUUCAUAAAAGUGCAG
>hsa-miR-590-3p	UAAUUUUAUGUAUAAGCUAGU
>hsa-miR-592	UUGUGUCAAUAUGCGAUGAUGU
>hsa-miR-597-5p	UGUGUCACUCGAUGACCACUGU
>hsa-miR-597-3p	UGGUUCUCUUGUGGCUCAAGCGU
>hsa-miR-598-3p	UACGUCAUCGUUGUCAUCGUCA
>hsa-miR-548a-5p	AAAAGUAAUUGCGAGUUUUACC
>hsa-miR-600	ACUUACAGACAAGAGCCUUGCUC
>hsa-miR-603	CACACACUGCAAUUACUUUUGC
>hsa-miR-605-5p	UAAAUCCCAUGGUGCCUUCUCCU
>hsa-miR-605-3p	AGAAGGCACUAUGAGAUUUAGA
>hsa-miR-607	GUUCAAAUCCAGAUCUAUAAC
>hsa-miR-610	UGAGCUAAAUGUGUGCUGGGA
>hsa-miR-612	GCUGGGCAGGGCUUCUGAGCUCCUU
>hsa-miR-615-3p	UCCGAGCCUGGGUCUCCCUCUU
>hsa-miR-616-5p	ACUCAAAACCCUUCAGUGACUU
>hsa-miR-616-3p	AGUCAUUGGAGGGUUUGAGCAG
>hsa-miR-548c-5p	AAAAGUAAUUGCGGUUUUUGCC
>hsa-miR-548c-3p	CAAAAAUCUCAAUUACUUUUGC
>hsa-miR-618	AAACUCUACUUGUCCUUCUGAGU
>hsa-miR-619-5p	GCUGGGAUUACAGGCAUGAGCC
>hsa-miR-624-5p	UAGUACCAGUACCUUGUGUUCA
>hsa-miR-624-3p	CACAAGGUAUUGGUAUUACCU
>hsa-miR-625-5p	AGGGGGAAAGUUCUAUAGUCC
>hsa-miR-625-3p	GACUAUAGAACUUUCCCCCUCA
>hsa-miR-626	AGCUGUCUGAAAAUGUCUU
>hsa-miR-627-5p	GUGAGUCUCUAAGAAAAGAGGA
>hsa-miR-627-3p	UCUUUUCUUUGAGACUCACU
>hsa-miR-628-5p	AUGCUGACAUAUUUACUAGAGG
>hsa-miR-628-3p	UCUAGUAAGAGUGGCAGUCGA
>hsa-miR-629-5p	UGGGUUUACGUUGGGAGAACU
>hsa-miR-629-3p	GUUCUCCCAACGUAAGCCCAGC
>hsa-miR-33b-5p	GUGCAUUGCUGUUGCAUUGC
>hsa-miR-33b-3p	CAGUGCCUCGGCAGUGCAGCCC
>hsa-miR-639	AUCGCUGCGGUUGCGAGCGCUGU
>hsa-miR-641	AAAGACAUAGGAUAGAGUCACCUC
>hsa-miR-642a-5p	GUCCCUCUCCAAAUGUGUCUUG
>hsa-miR-642a-3p	AGACACAUUUGGAGAGGGAACC
>hsa-miR-643	ACUUGUAUGCUAGCUCAGGUAG

>hsa-miR-651-5p	UUUAGGAUAAGCUUGACUUUUG
>hsa-miR-652-5p	CAACCCUAGGAGAGGGUGCCAUUCA
>hsa-miR-652-3p	AAUGGCGCCACUAGGGUUGUG
>hsa-miR-548d-5p	AAAAGUAAUUGUGGUUUUUGCC
>hsa-miR-548d-3p	CAAAAACCACAGUUUCUUUUGC
>hsa-miR-663a	AGGCGGGGCGCCGCGGGACCGC
>hsa-miR-449b-5p	AGGCAGUGUAUUGUUAGCUGGC
>hsa-miR-449b-3p	CAGCCACAACUACCCUGCCACU
>hsa-miR-653-5p	GUGUUGAAACAAUCUCUACUG
>hsa-miR-653-3p	UUCACUGGAGUUUGUUUCAAUA
>hsa-miR-411-5p	UAGUAGACCGUAUAGCGUACG
>hsa-miR-411-3p	UAUGUAACACGGUCCACUAACC
>hsa-miR-654-5p	UGGUGGGCCGCAGAACAUGUGC
>hsa-miR-654-3p	UAUGUCUGCUGACCAUCACCUU
>hsa-miR-655-5p	AGAGGUUAUCCGUGUUAUGUUC
>hsa-miR-655-3p	AUAAUACAUGGUUAACCUCUUU
>hsa-miR-656-5p	AGGUUGCCUGUGAGGUGUUCA
>hsa-miR-656-3p	AAUAUUAUACAGUCAACCUCU
>hsa-miR-549a	UGACAACUAUGGAUGAGCUCU
>hsa-miR-659-5p	AGGACCUUCCCUGAACCAAGGA
>hsa-miR-660-5p	UACCCAUUGCAUAUCGGAGUUG
>hsa-miR-660-3p	ACCUCCUGUGUGCAUGGAUUA
>hsa-miR-421	AUCAACAGACAUUAAUUGGGCGC
>hsa-miR-542-5p	UCGGGGAUCAUCAUGUCACGAGA
>hsa-miR-542-3p	UGUGACAGAUUGAUAACUGAAA
>hsa-miR-758-5p	GAUGGUUGACCAGAGAGCACAC
>hsa-miR-758-3p	UUUGUGACCUGGUCCACUAACC
>hsa-miR-1264	CAAGUCUUAUUUGAGCACCUGUU
>hsa-miR-671-5p	AGGAAGCCCUGGAGGGGCUGGAG
>hsa-miR-671-3p	UCCGGUUCUCAGGGCUCCACC
>hsa-miR-668-3p	UGUCACUCGGCUCGGCCCACUAC
>hsa-miR-550a-3-5p	AGUGCCUGAGGGAGUAAGAG
>hsa-miR-767-5p	UGCACCAUGGUUGUCUGAGCAUG
>hsa-miR-151b	UCGAGGAGCUCACAGUCU
>hsa-miR-320b	AAAAGCUGGGUUGAGAGGGCAA
>hsa-miR-320c	AAAAGCUGGGUUGAGAGGGU
>hsa-miR-1296-5p	UUAGGGCCCUGGCUCCAUCUCC
>hsa-miR-1296-3p	GAGUGGGGCUUCGACCCUAACC
>hsa-miR-1468-5p	CUCCGUUUGCCUGUUUCGCUG
>hsa-miR-1323	UCAAAACUGAGGGGCAUUUUCU
>hsa-miR-1271-5p	CUUGGCACCUAGCAAGCACUCA
>hsa-miR-1301-3p	UUGCAGCUGCCUGGGAGUGACUUC
>hsa-miR-454-5p	ACCCUAUCAAUAUUGUCUCUGC
>hsa-miR-454-3p	UAGUGCAAUAUUGCUUAUAGGGU
>hsa-miR-1185-5p	AGAGGAUACCCUUUGUAUGUU
>hsa-miR-1185-2-3p	AUAUACAGGGGGAGACUCUCAU
>hsa-miR-449c-5p	UAGGCAGUGUAUUGCUAGCGGCUGU
>hsa-miR-449c-3p	UUGCUAGUUGCACUCCUCUCUGU
>hsa-miR-1283	UCUACAAAGGAAAGCGCUUUCU
>hsa-miR-769-5p	UGAGACCUCUGGGUUCUGAGCU
>hsa-miR-769-3p	CUGGGAUCUCCGGGGUCUUGGUU
>hsa-miR-766-5p	AGGAGGAAUUGGUGCUGGUCUU
>hsa-miR-766-3p	ACUCCAGCCCCACAGCCUCAGC
>hsa-miR-378d	ACUGGACUUGGAGUCAGAAA
>hsa-miR-1185-1-3p	AUAUACAGGGGGAGACUCUUAU
>hsa-miR-802	CAGUAACAAAGAUUCAUCCUUGU
>hsa-miR-1298-5p	UUCAUUCGGCUGUCCAGAUGUA
>hsa-miR-765	UGGAGGAGAAGGAAGGUGAUG

>hsa-miR-770-5p UCCAGUACCACGUGUCAGGGCCA
>hsa-miR-675-5p UGGUGCGGAGAGGGCCCACAGUG
>hsa-miR-675-3p CUGUAUGCCCUCACCGCUCA
>hsa-miR-891a-5p UGCAACGAACCUGAGCCACUGA
>hsa-miR-300 UAUACAAGGGCAGACUCUCUCU
>hsa-miR-892a CACUGUGUCCUUUCUGCGUAG
>hsa-miR-450b-5p UUUUGCAAUAUGUUCCUGAAUA
>hsa-miR-450b-3p UUGGGAUCAUUUUGCAUCCAUA
>hsa-miR-874-5p CGGCCCCACGCACCAGGGUAAGA
>hsa-miR-874-3p CUGCCCUGGCCCGAGGGACCGA
>hsa-miR-890 UACUUGGAAAGGCAUCAGUUG
>hsa-miR-891b UGCAACUUACCUGAGUCAUUGA
>hsa-miR-888-5p UACUCAAAAAGCUGUCAGUCA
>hsa-miR-892b CACUGGCUCCUUUCUGGGUAGA
>hsa-miR-541-5p AAAGGAUUCUGCUGUCGGUCCCACU
>hsa-miR-541-3p UGGUGGGCACAGAAUCUGGACU
>hsa-miR-889-5p AAUGGCUGUCCGUAGUAUGGUC
>hsa-miR-889-3p UUAAUAUCGGACAACCAUUGU
>hsa-miR-876-5p UGGAUUUCUUUGUGAAUCACCA
>hsa-miR-876-3p UGGUGGUUUACAAAGUAAUUCA
>hsa-miR-708-5p AAGGAGCUUACAAUCUAGCUGGG
>hsa-miR-708-3p CAACUAGACUGUGAGCUUCUAG
>hsa-miR-147b GUGUGCGGAAAUGCUUCUGCUA
>hsa-miR-190b UGAUAUGUUUGAUAUUGGGUU
>hsa-miR-744-5p UGCGGGGCUAGGGCUAACAGCA
>hsa-miR-744-3p CUGUUGCCACUAACCUCAACCU
>hsa-miR-885-5p UCCAUUACACUACCCUGCCUCU
>hsa-miR-885-3p AGGCAGCGGGGUGUAGUGGAUA
>hsa-miR-877-5p GUAGAGGAGAUGGCGCAGGG
>hsa-miR-877-3p UCCUCUUCUCCCUCCUCCCAG
>hsa-miR-887-5p CUUGGGAGCCCUGUUAGACUC
>hsa-miR-887-3p GUGAACGGGCGCCAUCCCGAGG
>hsa-miR-665 ACCAGGAGGCUGAGGCCCCU
>hsa-miR-873-5p GCAGGAACUUGUGAGUCUCCU
>hsa-miR-873-3p GGAGACUGAUGAGUUCCCGGGA
>hsa-miR-543 AAACAUUCGCGGUGCACUUCUU
>hsa-miR-374b-5p AUAUAAUACAACCUGCUAAGUG
>hsa-miR-374b-3p CUUAGCAGGUUGUAUUAUCAUU
>hsa-miR-760 CGGCUCUGGGUCUGUGGGGA
>hsa-miR-301b-5p GCUCUGACGAGGUUGCACUACU
>hsa-miR-301b-3p CAGUGCAAUGAUAUUGUCAAAGC
>hsa-miR-216b-5p AAAUCUCUGCAGGCAAAUGUGA
>hsa-miR-509-3-5p UACUGCAGACGUGGCAAUCAUG
>hsa-miR-934 UGUCUACUACUGGAGACACUGG
>hsa-miR-936 ACAGUAGAGGGAGGAAUCGCAG
>hsa-miR-937-3p AUCCGCGCUCUGACUCUCUGCC
>hsa-miR-939-5p UGGGGAGCUGAGGCUCUGGGGGUG
>hsa-miR-939-3p CCCUGGGCCUCUGCUCCCCAG
>hsa-miR-940 AAGGCAGGGCCCCCGCUCCCC
>hsa-miR-941 CACCCGGCUGUGUGCACAUGUGC
>hsa-miR-942-5p UCUUCUCUGUUUUGGCCAUGUG
>hsa-miR-942-3p CACAUGGCCGAAACAGAGAAGU
>hsa-miR-943 CUGACUGUUGCCGUCCUCCAG
>hsa-miR-944 AAAUUAUUGUACAUCGGAUGAG
>hsa-miR-1179 AAGCAUUCUUUCAUUGGUUGG
>hsa-miR-1180-5p GGACCCACCCGGCCGGGAAUA
>hsa-miR-1180-3p UUUCCGGCUCGCGUGGGUGUGU
>hsa-miR-1226-5p GUGAGGGCAUGCAGGCCUGGAUGGGG

>hsa-miR-1226-3p	UCACCAGCCCUGUGUUCCCUAG
>hsa-miR-1228-3p	UCACACCUGCCUCGCCCCCC
>hsa-miR-1229-3p	CUCUCACCACUGCCCUCCCACAG
>hsa-miR-1234-3p	UCGGCCUGACCACCCACCCCAC
>hsa-miR-1237-3p	UCCUUCUGCUCCGUCCCCCAG
>hsa-miR-663b	GGUGGCCCGGCCGUGCCUGAGG
>hsa-miR-548e-5p	CAAAAGCAAUCGCGGUUUUUGC
>hsa-miR-548e-3p	AAAAACUGAGACUACUUUUGCA
>hsa-miR-548j-5p	AAAAGUAAUUGCGGUCUUUGGU
>hsa-miR-1285-5p	GAUCUCACUUUGUUGCCCAGG
>hsa-miR-1285-3p	UCUGGGCAACAAAGUGAGACCU
>hsa-miR-1286	UGCAGGACCAAGAUGAGCCCU
>hsa-miR-1287-5p	UGCUGGAUCAGUGGUUCGAGUC
>hsa-miR-1289	UGGAGUCCAGGAAUCUGCAUUUU
>hsa-miR-1290	UGGAUUUUUGGAUCAGGGA
>hsa-miR-1291	UGGCCCUGACUGAAGACCAGCAGU
>hsa-miR-548k	AAAAGUACUUGCGGAUUUUGCU
>hsa-miR-1293	UGGGUGGUCUGGAGAUUUGUGC
>hsa-miR-1294	UGUGAGGUUGGCAUUGUUGUCU
>hsa-miR-1295a	UUAGGCCGCAGAUCUGGGUGA
>hsa-miR-1297	UUCAAGUAAUUCAGGUG
>hsa-miR-1299	UUCUGGAAUUCUGUGUGAGGGA
>hsa-miR-548l	AAAAGUAUUUGCGGGUUUUGUC
>hsa-miR-1302	UUGGGACAUACUUAUGCUAAA
>hsa-miR-1303	UUUAGAGACGGGGUCUUGCUCU
>hsa-miR-1304-5p	UUUGAGGCUACAGUGAGAUGUG
>hsa-miR-1304-3p	UCUCACUGUAGCCUCGAACCCC
>hsa-miR-1305	UUUUCAACUCUAAUGGGAGAGA
>hsa-miR-1243	AACUGGAUCAAUUAUAGGAGUG
>hsa-miR-548f-3p	AAAAACUGUAAUUACUUUU
>hsa-miR-1245a	AAGUGAUCUAAAGGCCUACAU
>hsa-miR-1247-5p	ACCCGUCCCGUUCGUCCCCGGA
>hsa-miR-1247-3p	CCCCGGGAACGUCGAGACUGGAGC
>hsa-miR-1248	ACCUUCUUGUAUAAGCACUGUGCUAAA
>hsa-miR-1249-3p	ACGCCCUUCCCCCCCUUCUUCA
>hsa-miR-1250-5p	ACGGUGCUGGAUGUGGCCUUU
>hsa-miR-1254	AGCCUGGAAGCUGGAGCCUGCAGU
>hsa-miR-1255a	AGGAUGAGCAAAGAAAGUAGAUU
>hsa-miR-1256	AGGCAUUGACUUCUCACUAGCU
>hsa-miR-1260a	AUCCCACCUCUGCCACCA
>hsa-miR-548g-5p	UGCAAAAGUAAUUGCAGUUUUUG
>hsa-miR-548g-3p	AAAACUGUAAUUACUUUUGUAC
>hsa-miR-1261	AUGGAUAAGGCUUUGGCUU
>hsa-miR-1262	AUGGGUGAAUUUGUAGAAGGAU
>hsa-miR-1263	AUGGUACCCUGGCAUACUGAGU
>hsa-miR-548n	CAAAAGUAAUUGUGGAUUUUGU
>hsa-miR-548m	CAAAGGUAUUUGUGGUUUUUG
>hsa-miR-548o-3p	CCAAAACUGCAGUUACUUUUGC
>hsa-miR-1266-5p	CCUCAGGGCUGUAGAACAGGGCU
>hsa-miR-1267	CCUGUUGAAGUGUAAUCCCCA
>hsa-miR-1268a	CGGGCGUGGUGGUGGGGG
>hsa-miR-1270	CUGGAGAUAUGGAAGAGCUGUGU
>hsa-miR-548h-5p	AAAAGUAAUCGCGGUUUUUGUC
>hsa-miR-548h-3p	CAAAAACCGCAAUUACUUUUGCA
>hsa-miR-1275	GUGGGGGAGAGGCUGUC
>hsa-miR-1276	UAAAGAGCCCUGUGGAGACA
>hsa-miR-1277-5p	AAAUAUAUAUAUAUAUGUACGUAU
>hsa-miR-1277-3p	UACGUAGAUAUAUAUGUAUUUU

>hsa-miR-548p	UAGCAAAAACUGCAGUUACUUU
>hsa-miR-548i	AAAAGUAAUUGCGGAUUUUGCC
>hsa-miR-1278	UAGUACUGUGCAUAUCAUCUAU
>hsa-miR-1281	UCGCCUCCUCCUCUCCC
>hsa-miR-1282	UCGUUUGCCUUUUUCUGCUU
>hsa-miR-1284	UCUAUACAGACCCUGGCUUUUC
>hsa-miR-1292-5p	UGGGAACGGGUUCCGGCAGACGCUG
>hsa-miR-1252-5p	AGAAGGAAAUUGAAUUCAUUUA
>hsa-miR-1255b-5p	CGGAUGAGCAAAGAAAGUGGUU
>hsa-miR-664a-5p	ACUGGCUAGGGAAAAUGAUUGGAU
>hsa-miR-664a-3p	UAUUCAUUUAUCCCCAGCCUACA
>hsa-miR-1306-3p	ACGUUGGCUCUGGUGGUG
>hsa-miR-513b-5p	UUCACAAGGAGGUGUCAUUUAU
>hsa-miR-513b-3p	AAAUGUCACCUUUUUGAGAGGA
>hsa-miR-513c-5p	UUCUCAAGGAGGUGUCGUUUAU
>hsa-miR-513c-3p	UAAAUUUCACCUUUCUGAGAAGA
>hsa-miR-1197	UAGGACACAUGGUCUACUUCU
>hsa-miR-1469	CUCGGCGCGGGGCGCGGGCUCC
>hsa-miR-1537-5p	AGCUGUAAUUAGUCAGUUUUCU
>hsa-miR-1537-3p	AAAACCGUCUAGUUACAGUUGU
>hsa-miR-1538	CGGCCCGGGCUGCUGCUGUUCCU
>hsa-miR-320d	AAAAGCUGGGUUGAGAGGA
>hsa-miR-1827	UGAGGCAGUAGAUUGAAU
>hsa-miR-1910-5p	CCAGUCCUGUGCCUGCCGCCU
>hsa-miR-1911-5p	UGAGUACCGCCAUGUCUGUUGGG
>hsa-miR-1912	UACCCAGAGCAUGCAGUGUGAA
>hsa-miR-1913	UCUGCCCCCUCCGCUGCUGCCA
>hsa-miR-1972	UCAGGCCAGGCACAGUGGCUCA
>hsa-miR-1973	ACCGUGCAAAGGUAGCAUA
>hsa-miR-2114-3p	CGAGCCUCAAGCAAGGGACUU
>hsa-miR-2115-3p	CAUCAGAAUUCAUGGAGGCUAG
>hsa-miR-2116-5p	GGUUCUUAGCAUAGGAGGUCU
>hsa-miR-2116-3p	CCUCCCAUGCCAAGAACUCCC
>hsa-miR-548q	GCUGGUGCAAAAGUAAUGGCGG
>hsa-miR-2276-3p	UCUGCAAGUGUCAGAGGCGAGG
>hsa-miR-2278	GAGAGCAGUGUGUGUUGCCUGG
>hsa-miR-2681-5p	GUUUUACCACCUCCAGGAGACU
>hsa-miR-2681-3p	UAUCAUGGAGUUGGUAAAGCAC
>hsa-miR-2682-5p	CAGGCAGUGACUGUUCAGACGUC
>hsa-miR-3115	AUAUGGGUUUACUAGUUGGU
>hsa-miR-3116	UGCCUGGAACAUAGUAGGGACU
>hsa-miR-3117-5p	AGACACUAUACGAGUCAUAU
>hsa-miR-3117-3p	AUAGGACUCAUAUAGUGCCAG
>hsa-miR-3118	UGUGACUGCAUUAUGAAAAUUCU
>hsa-miR-3120-3p	CACAGCAAGUGUAGACAGGCA
>hsa-miR-3121-3p	UAAAUAGAGUAGGCAAAGGACA
>hsa-miR-3124-5p	UUCGCGGGCGAAGGCAAAGUC
>hsa-miR-548s	AUGGCCAAAACUGCAGUUAUUUU
>hsa-miR-3126-5p	UGAGGGACAGAUGCCAGAAGCA
>hsa-miR-3126-3p	CAUCUGGCAUCCGUCACACAGA
>hsa-miR-3127-5p	AUCAGGGCUUGUGGAAUGGGAAG
>hsa-miR-3128	UCUGGCAAGUAAAAAACUCUCAU
>hsa-miR-3129-5p	GCAGUAGUGUAGAGAUUGGUUU
>hsa-miR-3129-3p	AAACUAAUCUCUACACUGCUGC
>hsa-miR-3130-5p	UACCCAGUCUCCGGUGCAGCC
>hsa-miR-3130-3p	GCUGCACCGGAGACUGGGUAA
>hsa-miR-3133	UAAAGAACUCUUAAAACCCAAU
>hsa-miR-378b	ACUGGACUUGGAGGCAGAA

>hsa-miR-3134	UGAUGGAUAAAAGACUACAUAUU
>hsa-miR-3135a	UGCCUAGGCUGAGACUGCAGUG
>hsa-miR-466	AUACACAUACACGCAACACACAU
>hsa-miR-3136-5p	CUGACUGAAUAGGUAGGGUCAUU
>hsa-miR-3136-3p	UGGCCCAACCUAUUCAGUUAGU
>hsa-miR-544b	ACCUGAGGUUGUGCAUUUCUAA
>hsa-miR-3138	UGUGGACAGUGAGGUAGAGGGAGU
>hsa-miR-3139	UAGGAGCUCAACAGAUGCCUGUU
>hsa-miR-3140-5p	ACCUGAAUUACCAAAAGCUUU
>hsa-miR-3140-3p	AGCUUUUGGGAAUUCAGGUAGU
>hsa-miR-548t-5p	CAAAAGUGAUCGUGGUUUUUG
>hsa-miR-548t-3p	AAAAACCACAAUUACUUUUGCACCA
>hsa-miR-3143	AUAACAUUGUAAAGCGCUUCUUUCG
>hsa-miR-548u	CAAAGACUGCAAUUACUUUUGCG
>hsa-miR-3144-3p	AUAUACCUGUUCGGUCUCUUUA
>hsa-miR-3145-3p	AGAUAUUUUGAGUGUUUGGAAUUG
>hsa-miR-1273c	GGCGACAAAACGAGACCCUGUC
>hsa-miR-548v	AGCUACAGUUACUUUUGCACCA
>hsa-miR-3148	UGGAAAAAACUGGUGUGUGCUU
>hsa-miR-3149	UUUGUAUGGAUAUGUGUGUGUAU
>hsa-miR-3151-5p	GGUGGGGCAAUGGGAUCAGGU
>hsa-miR-3152-5p	AUUGCCUCUGUUCUAACACAAG
>hsa-miR-3074-5p	GUUCCUGCUGAACUGAGCCAG
>hsa-miR-3074-3p	GAUAUCAGCUCAGUAGGCACCG
>hsa-miR-3154	CAGAAGGGGAGUUGGGAGCAGA
>hsa-miR-3155a	CCAGGCUCUGCAGUGGGAACU
>hsa-miR-3157-5p	UUCAGCCAGGCUAGUGCAGUCU
>hsa-miR-3157-3p	CUGCCCUAGUCUAGCUGAAGCU
>hsa-miR-3158-5p	CCUGCAGAGAGGAAGCCCUUC
>hsa-miR-3158-3p	AAGGGCUUCCUCUCUGCAGGAC
>hsa-miR-3159	UAGGAUUACAAGUGUCGGCCAC
>hsa-miR-3160-3p	AGAGCUGAGACUAGAAAGCCCA
>hsa-miR-3161	CUGAUAAGAACAGAGGCCCAGAU
>hsa-miR-3162-3p	UCCCUACCCCUCCACUCCCCA
>hsa-miR-3164	UGUGACUUUAAGGGAAAUGGCG
>hsa-miR-3165	AGGUGGAUGCAAUGUGACCUCA
>hsa-miR-3166	CGCAGACAAUGCCUACUGGCCUA
>hsa-miR-1260b	AUCCCACCACUGCCACCAU
>hsa-miR-3167	AGGAUUUCAGAAAUACUGGUGU
>hsa-miR-3168	GAGUUCUACAGUCAGAC
>hsa-miR-3173-5p	UGCCCUGCCUGUUUUCUCCUUU
>hsa-miR-323b-5p	AGGUUGUCCGUGGUGAGUUCGCA
>hsa-miR-323b-3p	CCCAAUACACGGUCGACCUCUU
>hsa-miR-3174	UAGUGAGUUAGAGAUGCAGAGCC
>hsa-miR-3176	ACUGGCCUGGGACUACCGG
>hsa-miR-3177-3p	UGCACGGCACUGGGGACACGU
>hsa-miR-3179	AGAAGGGGUGAAAUUUAAACGU
>hsa-miR-3180-3p	UGGGGCGGAGCUUCCGGAGGCC
>hsa-miR-548w	AAAAGUAACUGCGGUUUUUGCCU
>hsa-miR-3182	GCUUCUGUAGUGUAGUC
>hsa-miR-3065-5p	UCAACAAAAUCACUGAUGCUGGA
>hsa-miR-3065-3p	UCAGCACCAGGAUAUUGUUGGAG
>hsa-miR-320e	AAAGCUGGGUUGAGAAGG
>hsa-miR-3190-3p	UGUGGAAGGUAGACGGCCAGAGA
>hsa-miR-3191-5p	CUCUCUGGCCGUCUACCUUCCA
>hsa-miR-3192-5p	UCUGGGAGGUUGUAGCAGUGGAA
>hsa-miR-3193	UCCUGCGUAGGAUCUGAGGAGU
>hsa-miR-3194-3p	AGCUCUGCUGCUCACUGGCAGU

>hsa-miR-3195 CGCGCCGGGCCCGGGUU
>hsa-miR-3196 CGGGGCGGCAGGGGCCUC
>hsa-miR-548x-5p UGCAAAAGUAAUUGCAGUUUUUG
>hsa-miR-548x-3p UAAAAACUGCAAUUACUUUC
>hsa-miR-3199 AGGGACUGCCUUAGGAGAAAGUU
>hsa-miR-3200-5p AAUCUGAGAAGGCGCACAAGGU
>hsa-miR-3200-3p CACCUUGCGCUACUCAGGUCUG
>hsa-miR-514b-5p UUCUCAAGAGGGAGGCAAUCAU
>hsa-miR-514b-3p AUUGACACCUCUGUGAGUGGA
>hsa-miR-3202 UGGAAGGGAGAAGAGCUUUAAU
>hsa-miR-1273d GAACCCAUGAGGUUGAGGCUGCAGU
>hsa-miR-378c ACUGGACUUGGAGUCAGAAGAGUGG
>hsa-miR-4301 UCCCACUACUUCACUUGUGA
>hsa-miR-4324 CCCUGAGACCCUAACCUUAA
>hsa-miR-4254 GCCUGGAGCUACUCCACCAUCUC
>hsa-miR-4326 UGUUCCUCUGUCUCCCAGAC
>hsa-miR-2355-5p AUCCCCAGAUACAAUGGACAA
>hsa-miR-2355-3p AUUGUCCUUGCUGUUUGGAGAU
>hsa-miR-4269 GCAGGCACAGACAGCCCUGGC
>hsa-miR-4270 UCAGGGAGUCAGGGGAGGGC
>hsa-miR-4284 GGGCUCACAUCACCCCAU
>hsa-miR-4286 ACCCCACUCCUGGUACC
>hsa-miR-4292 CCCCUGGGCCGGCCUUGG
>hsa-miR-500b-5p AAUCCUUGCUACCUGGGU
>hsa-miR-500b-3p GCACCCAGGCAAGGAUUCUG
>hsa-miR-3605-5p UGAGGAUGGAUAGCAAGGAAGCC
>hsa-miR-3605-3p CCUCCGUGUUACCUGUCCUCUAG
>hsa-miR-3606-5p UUAGUGAAGGCUAUUUUAAUU
>hsa-miR-3606-3p AAAAUUUCUUUCACUACUUAG
>hsa-miR-3609 CAAAGUGAUGAGUAAUACUGGCUG
>hsa-miR-3611 UUGUGAAGAAAGAAAUUCUUA
>hsa-miR-3612 AGGAGGCAUCUUGAGAAAUGGA
>hsa-miR-3613-5p UGUUGUACUUUUUUUUUUGUUC
>hsa-miR-3613-3p ACAAAAAAAAAAGCCCAACCCUUC
>hsa-miR-3614-3p UAGCCUUCAGAUCUUGGUGUUUU
>hsa-miR-3616-5p AUGAAGUGCACUCAUGAUAUGU
>hsa-miR-3616-3p CGAGGGCAUUUCAUGAUGCAGGC
>hsa-miR-3617-5p AAAGACAUAGUUGCAAGAUGGG
>hsa-miR-3617-3p CAUCAGCACCCUAUGUCCUUUCU
>hsa-miR-3619-5p UCAGCAGGCAGGCUGGUGCAGC
>hsa-miR-3619-3p GGGACCAUCCUGCCUGCUGUGG
>hsa-miR-23c AUCACAUUGCCAGUGAUUACCC
>hsa-miR-3622a-5p CAGGCACGGGAGCUCAGGUGAG
>hsa-miR-3622a-3p UCACCUGACCUCCCAUGCCUGU
>hsa-miR-3648 AGCCGCGGGGAUCGCCGAGGG
>hsa-miR-3653-3p CUAAGAAGUUGACUGAAG
>hsa-miR-3656 GGCGGGUGCGGGGGUGG
>hsa-miR-1273e UUGCUUGAACCCAGGAAGUGGA
>hsa-miR-3659 UGAGUGUUGUCUACGAGGGCA
>hsa-miR-3661 UGACCUGGGACUCGGACAGCUG
>hsa-miR-3663-5p GCUGGUCUGCGUGGUGCUCGG
>hsa-miR-3664-5p AACUCUGUCUUCACUCAUGAGU
>hsa-miR-3664-3p UCUCAGGAGUAAAGACAGAGUU
>hsa-miR-3667-3p ACCUUCCUCUCCAUGGGUCUUU
>hsa-miR-3675-5p UAUGGGGCUUCUGUAGAGAUUUC
>hsa-miR-3675-3p CAUCUCUAAGGAACUCCCCCAA
>hsa-miR-3677-3p CUCGUGGGCUCUGGCCACGGCC
>hsa-miR-3679-5p UGAGGAUAUGGCAGGGAAGGGGA

>hsa-miR-3679-3p	CUUCCCCCCAGUAAUCUUCAUC
>hsa-miR-3680-5p	GACUCACUCACAGGAUUGUGCA
>hsa-miR-3681-5p	UAGUGGAUGAUGCACUCUGUGC
>hsa-miR-3682-5p	CUACUUCUACCUGUGUUAUCAU
>hsa-miR-3684	UUAGACCUAGUACACGUCCUU
>hsa-miR-3687	CCCGGACAGGCGUUCGUGCGACGU
>hsa-miR-3688-3p	UAUGGAAAGACUUUGCCACUCU
>hsa-miR-3690	ACCUGGACCCAGCGUAGACAAAG
>hsa-miR-3691-5p	AGUGGAUGAUGGAGACUCGGUAC
>hsa-miR-3691-3p	ACCAAGUCUGCGUCAUCCUCUC
>hsa-miR-3692-3p	GUUCCACACUGACACUGCAGAAGU
>hsa-miR-3180	UGGGGCGGAGCUUCCGGAG
>hsa-miR-3908	GAGCAAUGUAGGUAGACUGUUU
>hsa-miR-3909	UGUCCUCUAGGGCCUGCAGUCU
>hsa-miR-3910	AAAGGCAUAAAACCAAGACA
>hsa-miR-3912-3p	UAACGCAUAAUAUGGACAUGU
>hsa-miR-3913-5p	UUUGGGACUGAUCUUGAUGUCU
>hsa-miR-3913-3p	AGACAUCAAGAUCAGUCCCAAA
>hsa-miR-3916	AAGAGGAAGAAAUGGCUGGUUCUCAG
>hsa-miR-3918	ACAGGGCCGCAGAUGGAGACU
>hsa-miR-3150b-5p	CAACCUCGAGGAUCUCCCCAGC
>hsa-miR-3150b-3p	UGAGGAGAUCGUCGAGGUUGG
>hsa-miR-3920	ACUGAUUAUCUUAACUCUCUGA
>hsa-miR-3921	UCUCUGAGUACCAUAUGCCUUGU
>hsa-miR-3922-5p	UCAAGGCCAGAGGUCCCACAGCA
>hsa-miR-3926	UGGCCAAAAAGCAGGCAGAGA
>hsa-miR-3927-3p	CAGGUAGAUAUUUGAUAGGCAU
>hsa-miR-676-5p	UCUUCAACCUCAGGACUUGCA
>hsa-miR-676-3p	CUGUCCUAAGGUUGUUGAGUU
>hsa-miR-3928-3p	GGAGGAACCUUGGAGCUUCGGC
>hsa-miR-3929	GAGGCUGAUGUGAGUAGACCACU
>hsa-miR-3934-5p	UCAGGUGUGGAAACUGAGGCAG
>hsa-miR-3934-3p	UGCUCAGGUUGCACAGCUGGGA
>hsa-miR-548y	AAAAGUAAUCACUGUUUUUGCC
>hsa-miR-3939	UACGCGCAGACCACAGGAUGUC
>hsa-miR-3940-3p	CAGCCCGGAUCCCAGCCCACUU
>hsa-miR-3942-5p	AAGCAAUACUGUUACCUGAAAU
>hsa-miR-3942-3p	UUUCAGAUAACAGUAUUACAU
>hsa-miR-3943	UAGCCCCCAGGCUUCACUUGGCG
>hsa-miR-3944-5p	UGUGCAGCAGGCCAACCGAGA
>hsa-miR-374c-5p	AUAAUACAACCUGCUAAGUGCU
>hsa-miR-374c-3p	CACUUAGCAGGUUGUAUUAUAU
>hsa-miR-642b-5p	GGUUCCCUCUCCAAAUGUGUCU
>hsa-miR-548z	CAAAAACCGCAAUUACUUUUGCA
>hsa-miR-548aa	AAAAACCACAAUUACUUUUGCACCA
>hsa-miR-548o-5p	AAAAGUAAUUGCGGUUUUUGCC
>hsa-miR-1268b	CGGGCGUGGUGGUGGGGGUG
>hsa-miR-378e	ACUGGACUUGGAGUCAGGA
>hsa-miR-548ab	AAAAGUAAUUGUGGAUUUUGCU
>hsa-miR-4417	GGUGGGCUUCCCGGAGGG
>hsa-miR-378f	ACUGGACUUGGAGCCAGAAG
>hsa-miR-4421	ACCUGUCUGUGGAAAGGAGCUA
>hsa-miR-4422	AAAAGCAUCAGGAAGUACCCA
>hsa-miR-4423-5p	AGUUGCCUUUUUGUUCCCAUGC
>hsa-miR-4423-3p	AUAGGCACCAAAAAGCAACAA
>hsa-miR-378g	ACUGGGCUUGGAGUCAGAAG
>hsa-miR-548ac	CAAAAACCGGCAAUUACUUUUG
>hsa-miR-4424	AGAGUUAACUCAAAAUGGACUA

>hsa-miR-4426	GAAGAUGGACGUACUUU
>hsa-miR-4428	CAAGGAGACGGGAACAUGGAGC
>hsa-miR-4429	AAAAGCUGGGCUGAGAGGCG
>hsa-miR-548ad-5p	AAAAGUAAUUGUGGUUUUUG
>hsa-miR-548ad-3p	GAAAACGACAAUGACUUUUGCA
>hsa-miR-548ae-3p	CAAAAACUGCAAUUACUUUCA
>hsa-miR-548ae-5p	AAAAGUAAUUGUGGUUUUUG
>hsa-miR-4440	UGUCGUGGGGCUUGCUGGCUUG
>hsa-miR-4443	UUGGAGGCGUGGGUUUU
>hsa-miR-4444	CUCGAGUUGGAAGAGGCG
>hsa-miR-4446-3p	CAGGGCUGGCAGUGACAUGGGU
>hsa-miR-4449	CGUCCCGGGGCUGCGCGAGGCA
>hsa-miR-548ag	AAAGGUAAUUGUGGUUUCUGC
>hsa-miR-548ah-5p	AAAAGUGAUUGCAGUGUUUG
>hsa-miR-548ah-3p	CAAAAACUGCAGUUACUUUUGC
>hsa-miR-4454	GGAUCCGAGUCACGGCACCA
>hsa-miR-4455	AGGGUGUGUGUGUUUUU
>hsa-miR-4458	AGAGGUAGGUGUGGAAGAA
>hsa-miR-4460	AUAGUGGUUGUGAAUUUACCUU
>hsa-miR-4461	GAUUGAGACUAGUAGGGCUAGGC
>hsa-miR-378h	ACUGGACUUGGUGUCAGAUGG
>hsa-miR-3135b	GGCUGGAGCGAGUGCAGUGGUG
>hsa-miR-548ai	AAAGGUAAUUGCAGUUUUUCCC
>hsa-miR-548aj-3p	UAAAAACUGCAAUUACUUUUA
>hsa-miR-548aj-5p	UGCAAAAGUAAUUGCAGUUUUUG
>hsa-miR-4467	UGGCGGCGGUAGUUAUGGGCUU
>hsa-miR-4470	UGGCAAACGUGGAAGCCGAGA
>hsa-miR-4473	CUAGUGCUCUCCGUUACAAGUA
>hsa-miR-4474-3p	UUGUGGCUGGUCAUGAGGCUAA
>hsa-miR-4477a	CUAUUAAGGACAUUUGUGAUUC
>hsa-miR-4477b	AUUAAGGACAUUUGUGAUUGAU
>hsa-miR-4478	GAGGCUGAGCUGAGGAG
>hsa-miR-3155b	CCAGGCUCUGCAGUGGGA
>hsa-miR-548ak	AAAAGUAACUGCGGUUUUUGA
>hsa-miR-4484	AAAAGGCGGGAGAAGCCCCA
>hsa-miR-4485-5p	ACCGCCUGCCCAGUGA
>hsa-miR-4485-3p	UAACGGCCGCGGUACCCUAA
>hsa-miR-4488	AGGGGGCGGGCUCCGGCG
>hsa-miR-4489	UGGGGCUAGUGAUGCAGGACG
>hsa-miR-548al	AACGGCAAUGACUUUUGUACCA
>hsa-miR-4491	AAUGUGGACUGGUGUGACCAAA
>hsa-miR-4492	GGGGCUGGGCGCGCGCC
>hsa-miR-4496	GAGGAAACUGAAGCUGAGAGGG
>hsa-miR-4497	CUCCGGGACGGCUGGGC
>hsa-miR-4498	UGGGCUGGCAGGGCAAGUGCUG
>hsa-miR-4419b	GAGGCUGAAGGAAGAUGG
>hsa-miR-4500	UGAGGUAGUAGUUUCUU
>hsa-miR-4504	UGUGACAAUAGAGAUGAACAUG
>hsa-miR-4508	GCGGGGCUGGGCGCGCG
>hsa-miR-4510	UGAGGGAGUAGGAUGUAUGGUU
>hsa-miR-4512	CAGGGCCUCACUGUAUCGCCCA
>hsa-miR-4516	GGGAGAAGGGUCGGGGC
>hsa-miR-4518	GCUCAGGGAUGAUAACUGUGCUGAGA
>hsa-miR-4520-3p	UUGGACAGAAAACACGCAGGAA
>hsa-miR-4521	GCUAAGGAAGUCCUGUGCUCAG
>hsa-miR-4524a-5p	AUAGCAGCAUGAACCUGUCUCA
>hsa-miR-4524a-3p	UGAGACAGGCUUAUGCUGCUAU
>hsa-miR-4526	GCUGACAGCAGGGCUGGCCGCU

>hsa-miR-4529-3p	AUUGGACUGCUGAUGGCCCGU
>hsa-miR-4532	CCCCGGGGAGCCCGGCG
>hsa-miR-378i	ACUGGACUAGGAGUCAGAAGG
>hsa-miR-548am-5p	AAAAGUAAUUGCGGUUUUUGCC
>hsa-miR-548am-3p	CAAAAACUGCAGUUACUUUUGU
>hsa-miR-1587	UUGGGCUGGGCUGGGUUGGG
>hsa-miR-4536-5p	UGUGGUAGAUAUAUGCACGAU
>hsa-miR-4536-3p	UCGUGCAUAUAUCUACCACAU
>hsa-miR-548an	AAAAGGCAUUGUGGUUUUUG
>hsa-miR-4632-3p	UGCCGCCCUCUCGCUGCUCUAG
>hsa-miR-4633-5p	AUAUGCCUGGCUAGCUCCUC
>hsa-miR-4633-3p	AGGAGCUAGCCAGGCAUAUGCA
>hsa-miR-4636	AACUCGUGUUCAAAGCCUUUAG
>hsa-miR-4638-5p	ACUCGGCUGCGGUGGACAAGU
>hsa-miR-4638-3p	CCUGGACACCGCUCAGCCGGCCG
>hsa-miR-4639-5p	UUGCUAAGUAGGCUGAGAUUGA
>hsa-miR-4644	UGGAGAGAGAAAAGAGACAGAAG
>hsa-miR-4645-3p	AGACAGUAGUUCUUGCCUGGUU
>hsa-miR-4646-3p	AUUGUCCCUCUCCCUUCCCAG
>hsa-miR-4649-3p	UCUGAGGCCUGCCUCUCCCCA
>hsa-miR-4650-5p	UCAGGCCUCUUUCUACCUU
>hsa-miR-4650-3p	AGGUAGAAUGAGGCCUGACAU
>hsa-miR-4659a-3p	UUUCUUCUUAGACAUGGCAACG
>hsa-miR-4661-5p	AACUAGCUCUGUGGAUCCUGAC
>hsa-miR-4661-3p	CAGGAUCCACAGAGCUAGUCCA
>hsa-miR-4662a-5p	UUAGCCAAUUGUCCAUCUUUAG
>hsa-miR-4662a-3p	AAAGAUAGACAAUUGGCUAAAU
>hsa-miR-4662b	AAAGAUGGACAAUUGGCUAAAU
>hsa-miR-4664-5p	UGGGGUGCCCACUCCGCAAGUU
>hsa-miR-4664-3p	CUUCCGGUCUGUGAGCCCCGUC
>hsa-miR-4668-5p	AGGGAAAAAAAAAAGGAUUUGUC
>hsa-miR-219b-5p	AGAUGUCCAGCCACAAUUCUCG
>hsa-miR-219b-3p	AGAAUUGCGUUUGGACAAUCAGU
>hsa-miR-4669	UGUGUCCGGGAAGUGGAGGAGG
>hsa-miR-4670-5p	AAGCGACCAUGAUGUAACUUCA
>hsa-miR-4670-3p	UGAAGUUACAUCAUGGUCGCUU
>hsa-miR-4672	UUACACAGCUGGACAGAGGCA
>hsa-miR-4676-3p	CACUGUUUCACCACUGGCUCUU
>hsa-miR-4677-5p	UUGUUCUUUGGUCUUUCAGCCA
>hsa-miR-4677-3p	UCUGUGAGACCAAAGAACUACU
>hsa-miR-4679	UCUGUGAUAGAGAUUCUUUGCU
>hsa-miR-4685-3p	UCUCCCUUCCUGCCCUGGCUAG
>hsa-miR-4687-5p	CAGCCCUCCUCCCGCACCCAAA
>hsa-miR-1343-3p	CUCCUGGGGCCCGCACUCUCGC
>hsa-miR-4690-3p	GCAGCCCAGCUGAGGCCUCUG
>hsa-miR-4695-3p	UGAUCUCACCGCUGCCUCCUUC
>hsa-miR-4696	UGCAAGACGGAUACUGUCAUCU
>hsa-miR-4697-3p	UGUCAGUGACUCCUGCCCCUUGGU
>hsa-miR-4699-5p	AGAAGAUUGCAGAGUAAGUUCC
>hsa-miR-4701-5p	UUGGCCACCACACCUACCCCUU
>hsa-miR-4704-3p	UCAGUCACAUAUCUAGUGUCUA
>hsa-miR-4705	UCAAUCACUUGGUAAUUGCUGU
>hsa-miR-4708-3p	AGCAAGGCGGCAUCUCUCUGAU
>hsa-miR-4709-5p	ACAACAGUGACUUGCUCUCCAA
>hsa-miR-203b-5p	UAGUGGUCCUAAACAUUUCACA
>hsa-miR-203b-3p	UUGAACUGUUAAGAACCACUGGA
>hsa-miR-4711-5p	UGCAUCAGGCCAGAAGACAUGAG
>hsa-miR-4713-5p	UUCUCCCACUACCAGGCUCCCA

>hsa-miR-4715-5p	AAGUUGGCUGCAGUUAAGGUGG
>hsa-miR-3529-5p	AGGUAGACUGGGAUUUGUUGUU
>hsa-miR-4717-3p	ACACAUGGGUGGCUGUGGCCU
>hsa-miR-4520-2-3p	UUUGGACAGAAAACACGCAGGU
>hsa-miR-451b	UAGCAAGAGAACCAUUACCAUU
>hsa-miR-4725-3p	UGGGGAAGGCGUCAGUGUCGGG
>hsa-miR-4728-3p	CAUGCUGACCUCCCUCCUGCCCCAG
>hsa-miR-4730	CUGGCGGAGCCCAUUCCAUGCCA
>hsa-miR-4731-5p	UGCUGGGGGCCACAUGAGUGUG
>hsa-miR-4731-3p	CACACAAGUGGCCCCCAACACU
>hsa-miR-4732-5p	UGUAGAGCAGGGAGCAGGAAGCU
>hsa-miR-4735-5p	CCUAAUUUGAACACCUUCGGUA
>hsa-miR-4737	AUGCGAGGAUGCUGACAGUG
>hsa-miR-3064-5p	UCUGGCUGUUGUGGUGUGCAA
>hsa-miR-3064-3p	UUGCCACACUGCAACACCUUACA
>hsa-miR-4738-3p	UGAAACUGGAGCGCCUGGAGGA
>hsa-miR-4741	CGGGCUGUCCGGAGGGGUCGGCU
>hsa-miR-4742-5p	UCAGGCAAAGGGAUAUUUACAGA
>hsa-miR-4742-3p	UCUGUAUUCUCCUUUGCCUGCAG
>hsa-miR-4743-3p	UUUCUGUCUUUUCUGGUCCAG
>hsa-miR-3591-5p	UUUAGUGUGAUAAUGGCGUUUGA
>hsa-miR-4746-5p	CCGGUCCCAGGAGAACCUGCAGA
>hsa-miR-4747-3p	AAGGCCCGGGCUUUCCUCCCAG
>hsa-miR-4749-5p	UGCGGGGACAGGCCAGGGCAUC
>hsa-miR-4753-3p	UUCUCUUUCUUUAGCCUUGUGU
>hsa-miR-371b-5p	ACUCAAAAGAUGGCGGCACUUU
>hsa-miR-4754	AUGCGGACCUGGGUUAGCGGAGU
>hsa-miR-4755-3p	AGCCAGGCUCUGAAGGGAAAGU
>hsa-miR-499b-5p	ACAGACUUGCUGUGAUGUUCA
>hsa-miR-4757-3p	CAUGACGUCACAGAGGCUUCGC
>hsa-miR-4759	UAGGACUAGAUGUUGGAAUUA
>hsa-miR-4761-5p	ACAAGGUGUGCAUGCCUGACC
>hsa-miR-4761-3p	GAGGGCAUGCGCACUUUGUCC
>hsa-miR-4763-5p	CGCCUGCCCAGCCCUCCUGCU
>hsa-miR-4766-3p	AUAGCAAUUGCUCUUUUGGAA
>hsa-miR-4767	CGCGGGCGCUCCUGGCCGCCGCC
>hsa-miR-4768-5p	AUUCUCUCUGGAUCCCAUGGAU
>hsa-miR-4772-5p	UGAUCAGGCAAAAUUGCAGACU
>hsa-miR-4772-3p	CCUGCAACUUUGCCUGAUCAGA
>hsa-miR-4773	CAGAACAGGAGCAUAGAAAGGC
>hsa-miR-4775	UUAAUUUUUUGUUUCGGUCACU
>hsa-miR-4777-3p	AUACCUCAUCUAGAAUGCUGUA
>hsa-miR-4436b-3p	CAGGGCAGGAAGAAGUGGACAA
>hsa-miR-4781-5p	UAGCGGGGAUUCCAAUAUUGG
>hsa-miR-4781-3p	AAUGUUGGAAUCCUCGCUAGAG
>hsa-miR-4782-5p	UUCUGGAUAUGAAGACAAUCAA
>hsa-miR-4785	AGAGUCGGCGACGCCGCCAGC
>hsa-miR-1245b-5p	UAGGCCUUUAGAUCACUUAAA
>hsa-miR-1245b-3p	UCAGAUGAUCUAAAGGCCUAUA
>hsa-miR-2467-5p	UGAGGCUCUGUUAGCCUUGGCUC
>hsa-miR-4786-5p	UGAGACCAGGACUGGAUGCACC
>hsa-miR-4787-5p	GCGGGGGUGGCGGCGGCAUCCC
>hsa-miR-4791	UGGAUAUGAUGACUGAAA
>hsa-miR-4792	CGGUGAGCGCUCGCUGGC
>hsa-miR-4795-5p	AGAAGUGGCUAAUAAUAUUGA
>hsa-miR-4795-3p	AUAUUAUUAGCCACUUCUGGAU
>hsa-miR-4796-5p	UGUCUAUACUCUGUCACUUUAC
>hsa-miR-4796-3p	UAAAGUGGCAGAGUAUAGACAC

>hsa-miR-4797-5p	GACAGAGUGCCACUUACUGAA
>hsa-miR-4797-3p	UCUCAGUAAGUGGCACUCUGU
>hsa-miR-4798-5p	UUCGGUAUACUUUGUGAAUUGG
>hsa-miR-4798-3p	AACUCACGAAGUAUACCGAAGU
>hsa-miR-4799-5p	AUCUAAAUGCAGCAUGCCAGUC
>hsa-miR-4799-3p	ACUGGCAUGCUGCAUUUAUAUA
>hsa-miR-4800-3p	CAUCCGUCCGUCUGUCCAC
>hsa-miR-4802-5p	UAUGGAGGUUCUAGACCAUGUU
>hsa-miR-4802-3p	UACAUGGAUGGAAACCUUCAAGC
>hsa-miR-4803	UAACAUAAUAGUGUGGAUUGA
>hsa-miR-4804-5p	UUGGACGGUAAGGUUAAGCAA
>hsa-miR-4804-3p	UGCUUAACCUUGCCCUCGAAA
>hsa-miR-4999-5p	UGCUGUAUUGUCAGGUAGUGA
>hsa-miR-4999-3p	UCACUACCUGACAAUACAGU
>hsa-miR-5000-5p	CAGUUCAGAAGUGUUCCUGAGU
>hsa-miR-5000-3p	UCAGGACACUUCUGAACUUGGA
>hsa-miR-5001-3p	UUCUGCCUCUGUCCAGGUCCUU
>hsa-miR-5002-5p	AAUUUGGUUUCUGAGGCACUUAGU
>hsa-miR-5003-5p	UCACAACAACCUUGCAGGGUAGA
>hsa-miR-5003-3p	UACUUUUCUAGGUUGUUGGGG
>hsa-miR-5004-3p	CUUGGAUUUUCCUGGGCCUCAG
>hsa-miR-5006-3p	UUUCCCUUUCCAUCCUGGCAG
>hsa-miR-548ap-5p	AAAAGUAAUUGCGGUCUUU
>hsa-miR-548ap-3p	AAAAACCACAAUUACUUUU
>hsa-miR-5008-5p	UGAGGCCCUUGGGGCACAGUGG
>hsa-miR-5009-5p	UUGGACUUUUUCAGAUUUGGGGAU
>hsa-miR-5010-5p	AGGGGGAUGGCAGAGCAAAAUU
>hsa-miR-5010-3p	UUUUGUGUCUCCCAUUCCCCAG
>hsa-miR-5087	GGGUUUGUAGCUUUGCUGGCAUG
>hsa-miR-5088-5p	CAGGGCUCAGGGAUUGGAUGGAGG
>hsa-miR-5088-3p	UCCCUUCUUCCUGGGCCCUCA
>hsa-miR-5089-5p	GUGGGAUUUCUGAGUAGCAUC
>hsa-miR-5089-3p	AUGCUACUCGGAAAUCCCACUGA
>hsa-miR-5091	ACGGAGACGACAAGACUGUGCUG
>hsa-miR-5092	AAUCCACGCUGAGCUUGGCAUC
>hsa-miR-5094	AAUCAGUGAAUGCCUUGAACCU
>hsa-miR-5095	UUACAGGCGUGAACCACCGCG
>hsa-miR-1273f	GGAGAUGGAGGUUGCAGUG
>hsa-miR-1273g-5p	GGUGGUUGAGGCUGCAGUAAGU
>hsa-miR-1273g-3p	ACCACUGCACUCCAGCCUGAG
>hsa-miR-5096	GUUUCACCAUGUUGGUCAGGC
>hsa-miR-5187-5p	UGGGAUGAGGGAUUGAAGUGGA
>hsa-miR-5187-3p	ACUGAAUCCUCUUUUCCUCAG
>hsa-miR-5188	AAUCGGACCCAUUUAAACCGGAG
>hsa-miR-5193	UCCUCCUCUACCUCAUCCCAGU
>hsa-miR-5196-3p	UCAUCCUCGUCUCCCUCCCAG
>hsa-miR-5571-3p	GUCCUAGGAGGCUCCUCUG
>hsa-miR-5100	UUCAGAUCCCAGCGGUGCCUCU
>hsa-miR-548aq-5p	GAAAGUAAUUGCUGUUUUUGCC
>hsa-miR-548aq-3p	CAAAAACUGCAAUUACUUUUGC
>hsa-miR-548ar-5p	AAAAGUAAUUGCAGUUUUUGC
>hsa-miR-548ar-3p	UAAAACUGCAGUUAUUUUUGC
>hsa-miR-548as-5p	AAAAGUAAUUGCGGGUUUUGCC
>hsa-miR-5579-5p	UAUGGUACUCCUUAAGCUAAC
>hsa-miR-5579-3p	UUAGCUUAAGGAGUACCAGAUC
>hsa-miR-664b-3p	UUCAUUUGCCUCCCAGCCUACA
>hsa-miR-5581-3p	UUCCAUGCCUCCUAGAAGUUCC
>hsa-miR-548at-5p	AAAAGUUAUUGCGGUUUUGGCU

>hsa-miR-5582-5p	UAGGCACACUUAAAGUUAUAGC
>hsa-miR-5585-5p	UGAAGUACCAGCUACUCGAGAG
>hsa-miR-5585-3p	CUGAAUAGCUGGGACUACAGGU
>hsa-miR-5586-5p	UAUCCAGCUUGUUACUAUAUGC
>hsa-miR-5587-3p	GCCCCGGGCAGUGUGAUCAUC
>hsa-miR-548au-5p	AAAAGUAAUUGCGGUUUUUGC
>hsa-miR-548au-3p	UGGCAGUUACUUUUGCACCAG
>hsa-miR-1295b-3p	AAUAGGCCACGGAUCUGGGCAA
>hsa-miR-5588-5p	ACUGGCAUUAGUGGGACUUUU
>hsa-miR-5588-3p	AAGUCCCACUAAUGCCAGC
>hsa-miR-548av-5p	AAAAGUACUUGCGGAUUU
>hsa-miR-548av-3p	AAAACUGCAGUUACUUUUGC
>hsa-miR-5680	GAGAAAUGCUGGACUAAUCUGC
>hsa-miR-548aw	GUGCAAAAGUCAUCACGGUU
>hsa-miR-5683	UACAGAUGCAGAUUCUCUGACUUC
>hsa-miR-5684	AACUCUAGCCUGAGCAACAG
>hsa-miR-5687	UUAGAACGUUUUAGGGUCAAAU
>hsa-miR-5689	AGCAUACACCUGUAGUCCUAGA
>hsa-miR-5690	UCAGCUACUACCUCUAUUAGG
>hsa-miR-5691	UUGCUCUGAGCUCCGAGAAAGC
>hsa-miR-5693	GCAGUGGCUCUGAAAUGAACUC
>hsa-miR-5695	ACUCCAAGAAGAAUCUAGACAG
>hsa-miR-5696	CUCAUUUAAGUAGUCUGAUGCC
>hsa-miR-5699-5p	UGCCCCAACAAGGAAGGACAAG
>hsa-miR-5699-3p	UCCUGUCUUUCCUUGUUGGAGC
>hsa-miR-5701	UUAUUGUCACGUUCUGAUU
>hsa-miR-5703	AGGAGAAGUCGGGAAGGU
>hsa-miR-6087	UGAGGCGGGGGGGCGAGC
>hsa-miR-6130	UGAGGGAGUGGAUUGUAUG
>hsa-miR-6131	GGCUGGUCAGAUGGGAGUG
>hsa-miR-6132	AGCAGGGCUGGGGAUUGCA
>hsa-miR-6134	UGAGGUGGUAGGAUGUAGA
>hsa-miR-6499-5p	UCGGGCGCAAGAGCACUGCAGU
>hsa-miR-548ay-5p	AAAAGUAAUUGUGGUUUUUGC
>hsa-miR-548ay-3p	CAAAACCGCGAUUACUCUUGCA
>hsa-miR-6500-3p	ACACUUGUUGGGAUGACCUGC
>hsa-miR-548az-5p	CAAAAGUGAUUGUGGUUUUUGC
>hsa-miR-548az-3p	AAAAACUGCAAUCACUUUUGC
>hsa-miR-6503-5p	AGGUCUGCAUUCAAAUCCCCAGA
>hsa-miR-6503-3p	GGGACUAGGAUGCAGACCUCC
>hsa-miR-6504-5p	UCUGGCUGUGCUGUAAUGCAG
>hsa-miR-6505-5p	UUGGAAUAGGGGAUAUCUCAGC
>hsa-miR-6506-5p	ACUGGGAUGUCACUGAAUAUGGU
>hsa-miR-6507-5p	GAAGAAUAGGAGGGACUUUGU
>hsa-miR-6508-3p	UGGGCCAUGCAUUUCUAGAACU
>hsa-miR-6509-5p	AUUAGGUAGUGGCAGUGGAAC
>hsa-miR-6509-3p	UUCCACUGCCACUACCUAAUUU
>hsa-miR-6510-3p	CACCGACUCUGUCUCCUGCAG
>hsa-miR-6511a-5p	CAGGCAGAAGUGGGGCUGACAGG
>hsa-miR-6511a-3p	CCUCACCAUCCCUUCUGCCUGC
>hsa-miR-6512-5p	UACCAUUAGAAGAGCUGGAAGA
>hsa-miR-6512-3p	UUCCAGCCCUUCUAAUGGUAGG
>hsa-miR-6513-5p	UUUGGGAUUGACGCCACAUGUCU
>hsa-miR-6513-3p	UCAAGUGUCAUCUGUCCCUAG
>hsa-miR-6514-5p	UAUGGAGUGGACUUUCAGCUGGC
>hsa-miR-6514-3p	CUGCCUGUUCUUCCACUCCAG
>hsa-miR-6515-5p	UUGGAGGGUGUGGAAGACAUC
>hsa-miR-6716-3p	UCCGAACUCUCCAUUCCUCUGC

>hsa-miR-6511b-5p	CUGCAGGCAGAAGUGGGGCUGACA
>hsa-miR-6511b-3p	CCUCACCACCCCUUCUGCCUGCA
>hsa-miR-6718-5p	UAGUGGUCAGAGGGCUUAUGA
>hsa-miR-6720-5p	UUCCAGCCCUGGUAGGCGCCGCG
>hsa-miR-6720-3p	CGCGCCUGCAGGAACUGGUAGA
>hsa-miR-6723-5p	AUAGUCCGAGUAACGUCGGGGC
>hsa-miR-6724-5p	CUGGGCCCGCGGCGGGCGUGGGG
>hsa-miR-892c-3p	CACUGUUUCCUUUCUGAGUGGA
>hsa-miR-6727-5p	CUCGGGGCAGGCGGCUGGGAGCG
>hsa-miR-6730-3p	CCUGACACCCCAUCUGCCCUCA
>hsa-miR-6731-5p	UGGGAGAGCAGGGUAUUGUGGA
>hsa-miR-6731-3p	UCUAUUCCCCACUCUCCCCAG
>hsa-miR-6733-5p	UGGGAAAGACAAACUCAGAGUU
>hsa-miR-6733-3p	UCAGUGUCUGGAUUUCCUAG
>hsa-miR-6734-5p	UUGAGGGGAGAAUGAGGUGGAGA
>hsa-miR-6734-3p	CCCUUCCCUCACUCUUCUCUCAG
>hsa-miR-6735-5p	CAGGGCAGAGGGCACAGGAAUCUGA
>hsa-miR-6735-3p	AGGCCUGUGGCUCCUCCCUCAG
>hsa-miR-6736-5p	CUGGGUGAGGGCAUCUGUGGU
>hsa-miR-6739-5p	UGGGAAAGAGAAAGAACAAGUA
>hsa-miR-6741-3p	UCGGCUCUCUCCCUCACCCUAG
>hsa-miR-6747-3p	UCCUGCCUUCCUCUGCACCAG
>hsa-miR-6751-3p	ACUGAGCCUCUCUCUCUCCAG
>hsa-miR-6754-3p	UCUUCACCUGCCUCUGCCUGCA
>hsa-miR-6757-5p	UAGGGAUGGGAGGCCAGGAUGA
>hsa-miR-6758-5p	UAGAGAGGGGAAGGAUGUGAUGU
>hsa-miR-6761-5p	UCUGAGAGAGCUCGAUGGCAG
>hsa-miR-6761-3p	UCCUACGCUGCUCUCUCACUCC
>hsa-miR-6764-5p	UCCCAGGGUCUGGUCAGAGUUG
>hsa-miR-6765-3p	UCACCUGGCUGGCCCGCCCAG
>hsa-miR-6766-3p	UGAUUGUCUUCCCCCACCCUCA
>hsa-miR-6769a-3p	GAGCCCCUCUCUGCUCUCCAG
>hsa-miR-6770-5p	UGAGAAGGCACAGCUUGCACGUGA
>hsa-miR-6770-3p	CUGGCGGCUGUGUCUUCACAG
>hsa-miR-6771-5p	CUCGGGAGGGCAUGGGCCAGGC
>hsa-miR-6772-5p	UGGGUGUAGGCUGGAGCUGAGG
>hsa-miR-6772-3p	UUGCUCCUGACUCUGUGCCCACA
>hsa-miR-6777-5p	ACGGGGAGUCAGGCAGUGGUGGA
>hsa-miR-6780a-5p	UUGGGAGGGAAGACAGCUGGAGA
>hsa-miR-6786-3p	UGACGCCCCUUCUGAUUCUGCCU
>hsa-miR-6787-3p	UCUCAGCUGCUGCCCUCUCCAG
>hsa-miR-6788-5p	CUGGGAGAAGAGUGGUGAAGA
>hsa-miR-6788-3p	UUCGCCACUUCCCUCCCUGCAG
>hsa-miR-6790-3p	CGACCUCGGCGACCCCUCACU
>hsa-miR-6797-3p	UGCAUGACCCUUCCCUCCCCAC
>hsa-miR-6800-3p	CACCUCUCCUGGCAUCGCCCC
>hsa-miR-6802-5p	CUAGGUGGGGGGCUUGAAGC
>hsa-miR-6802-3p	UUCACCCCUCUCACCUAAGCAG
>hsa-miR-6804-3p	CGCACCUGCCUCUCACCCACAG
>hsa-miR-6809-3p	CUUCUCUUCUCUCCUUCCCAG
>hsa-miR-6811-5p	AUGCAGGCCUGUGUACAGCACU
>hsa-miR-6813-5p	CAGGGGCUGGGGUUUCAGGUUCU
>hsa-miR-6813-3p	AACCUUGGCCCCUCUCCCCAG
>hsa-miR-6815-5p	UAGGUGGCGCCGGAGGAGUCAUU
>hsa-miR-6818-5p	UUGUGUGAGUACAGAGAGCAUC
>hsa-miR-6818-3p	UUGUCUCUUGUUCCUCACACAG
>hsa-miR-6820-5p	UGCGGCAGAGCUGGGGUCA
>hsa-miR-6820-3p	UGUGACUUCUCCCCUGCCACAG

>hsa-miR-6822-5p	CAGGGAACCAGUUGGGGCUU
>hsa-miR-6826-5p	UCAAUAGGAAAGAGGUGGGACCU
>hsa-miR-6827-5p	UGGGAGCCAUGAGGGUCUGUGC
>hsa-miR-6780b-5p	UGGGGAAGGCUUGGCAGGGAAGA
>hsa-miR-6836-5p	CGCAGGGCCCUGGCGCAGGCAU
>hsa-miR-6837-5p	ACCAGGGCCAGCAGGGAAUGU
>hsa-miR-6837-3p	CCUUCACUGUGACUCUGCUGCAG
>hsa-miR-6838-5p	AAGCAGCAGUGGCAAGACUCCU
>hsa-miR-6839-5p	UCUGGAUUGAAGAGACGACCCA
>hsa-miR-6842-5p	UGGGGGUGGUCUCUAGCCAAGG
>hsa-miR-6842-3p	UUGGCUGGUCUCUGCUCCGCAG
>hsa-miR-6844	UUCUUUGUUUUUAAUUCACAG
>hsa-miR-6851-5p	AGGAGGUGGUACUAGGGGCCAGC
>hsa-miR-6852-5p	CCCUGGGGUUCUGAGGACAUG
>hsa-miR-6854-5p	AAGCUCAGGUUUGAGAACUGCUGA
>hsa-miR-6769b-3p	CCCUCUCUGUCCCACCCAUAG
>hsa-miR-6861-3p	UGGACCUCUCCUCCCCAG
>hsa-miR-6862-5p	CGGGCAUGCUGGGAGAGACUUU
>hsa-miR-6865-5p	UAGGUGGCAGAGGAGGGACUUCA
>hsa-miR-6866-5p	UUAGAGGCUGGAAUAGAGAUUCU
>hsa-miR-6870-3p	GCUCAUCCCCAUCUCCUUUCAG
>hsa-miR-6871-3p	CAGCACCCUGUGGCUCCCACAG
>hsa-miR-6873-3p	UUCUCUCUGUCUUUCUCUCUCAG
>hsa-miR-6874-3p	CAGUUCUGCUGUUCUGACUCUAG
>hsa-miR-6875-5p	UGAGGGACCCAGGACAGGAGA
>hsa-miR-6875-3p	AUUCUUCCUGCCCUGGCUCCAU
>hsa-miR-6876-5p	CAGGAAGGAGACAGGCAGUUCA
>hsa-miR-6877-5p	AGGGCCGAAGGGUGGAAGCUGC
>hsa-miR-6878-5p	AGGGAGAAAGCUAGAAGCUGAAG
>hsa-miR-6880-5p	UGGUGGAGGAAGAGGGCAGCUC
>hsa-miR-6882-5p	UACAAGUCAGGAGCUGAAGCAG
>hsa-miR-6883-3p	UUCCCUAUCUCACUCUCCUCAG
>hsa-miR-6884-5p	AGAGGCUGAGAAGGUGAUGUUG
>hsa-miR-6884-3p	CCCAUCACCUUUCCGUCUCCCCU
>hsa-miR-6889-5p	UCGGGGAGUCUGGGGUCCGGAAU
>hsa-miR-6890-3p	CCACUGCCUAUGCCCCACAG
>hsa-miR-6892-5p	GUAAGGGACCGGAGAGUAGGA
>hsa-miR-6894-3p	UUGCCUGCCCUCUUCCUCCAG
>hsa-miR-7109-3p	CAAGCCUCUCCUGCCCUUCCAG
>hsa-miR-7110-3p	UCUCUCUCCCACUUCCCUGCAG
>hsa-miR-7112-5p	ACGGGCAGGGCAGUGCACCCUG
>hsa-miR-7113-3p	CCUCCCUGCCCGCCUCUCUGCAG
>hsa-miR-7151-3p	CUACAGGCUGGAAUGGGCUCA
>hsa-miR-7155-3p	UGGCCCAAGACCUCAGACC
>hsa-miR-7641	UUGAUCUCGGAAGCUAAGC
>hsa-miR-7704	CGGGGUCGGCGGCGACGUG
>hsa-miR-7705	AAUAGCUCAGAAUGUCAGUUCUG
>hsa-miR-7706	UGAAGCGCCUGUGCUCUGCCGAGA
>hsa-miR-4433b-5p	AUGUCCCACCCCCACUCCUGU
>hsa-miR-1273h-5p	CUGGGAGGUCAAGGCUGCAGU
>hsa-miR-1273h-3p	CUGCAGACUCGACCUCCCAGGC
>hsa-miR-6516-5p	UUUGCAGUAACAGGUGUGAGCA
>hsa-miR-6516-3p	AUCAUGUAUGAUACUGCAAACA
>hsa-miR-7846-3p	CAGCGGAGCCUGGAGAGAAGG
>hsa-miR-7849-3p	GACAAUUGUUGAUCUUGGGCCU
>hsa-miR-7852-3p	UAUGUAGUAGUCAAAGGCAUUU
>hsa-miR-7854-3p	UGAGGUGACCGCAGAUGGGAA
>hsa-miR-548ba	AAAGGUAACUGUGAUUUUUGCU

>hsa-miR-7973	UGUGACCCUAGAAUAAUUAC
>hsa-miR-7974	AGGCUGUGAUGCUCUCCUGAGCCC
>hsa-miR-7975	AUCCUAGUCACGGCACCA
>hsa-miR-7976	UGCCCUGAGACUUUUGCUC
>hsa-miR-7977	UUCCCAGCCAACGCACCA
>hsa-miR-8485	CACACACACACACACACGUAU
>hsa-novel-1a	CCUGUCUGAGCGUCGCU
>hsa-novel-1b	CCUGUCUGAGCGUCGCU
>hsa-novel-2	UGUGUAACCACUCACCU
>hsa-novel-3a	GAGCUUGACUCUAGUCU
>hsa-novel-3b	GAGCUUGACUCUAGUCU
>hsa-novel-4	GUUUCCGUAGUGUAGUG
>hsa-novel-5	CAGCCUGAGCAACAUAGC
>hsa-novel-6	AUCUAUUGAAAGUCAGC
>hsa-novel-7a	UGAGGCAGGAGAAUCGCUU
>hsa-novel-7b	UGAGGCAGGAGAAUCGCUU
>hsa-novel-7c	UGAGGCAGGAGAAUCGCUU
>hsa-novel-8	UUCACAGUGGCCAAGUUU
>hsa-novel-9	CAGAAACCAGAAUUACUUUUGC
>hsa-novel-10a	GAGGCAGGAGAAUCGCUU
>hsa-novel-10b	GAGGCAGGAGAAUCGCUU
>hsa-novel-10c	GAGGCAGGAGAAUCGCUU
>hsa-novel-10d	GAGGCAGGAGAAUCGCUU
>hsa-novel-11a	CCAGCUACUCGGGAGGCU
>hsa-novel-11b	CCAGCUACUCGGGAGGCU
>hsa-novel-12	UAGCGAGACCCCGUCUCUA
>hsa-novel-13	GCGAGGCGGGGCUGGGC
>hsa-novel-14	AAAAGUAACUGUGGUUUUUGCC
>hsa-novel-15a	CAAAAACCAAAAUUACUUUUGC
>hsa-novel-15b	CAAAAACCAAAAUUACUUUUGC
>hsa-novel-16	AGUUCUGGGCUGUAGUG
>hsa-novel-17	AAAAGUAAUCGUGGAUUUUGCU
>hsa-novel-18	AGAAUCUGUUGGUAAAGCCUCU
>hsa-novel-19	CAAAAACCGUAAUUACUUUUGU
>hsa-novel-20	UAGAAGCACAGAAAUAUUAG
>hsa-novel-21a	UGGGAUUACAGGCGUGAGCCA
>hsa-novel-21b	UGGGAUUACAGGCGUGAGCCA
>hsa-novel-22	CGUGCUGAUCAGUAGUGG
>hsa-novel-23	UAUGUGCCUAGUGGCUGCUGUCU
>hsa-novel-24a	ACUCCAGCCUGGGCAAC
>hsa-novel-24b	ACUCCAGCCUGGGCAAC
>hsa-novel-24c	ACUCCAGCCUGGGCAAC
>hsa-novel-24d	ACUCCAGCCUGGGCAAC
>hsa-novel-25	GUGCAAAAGUCAUUGUGGUUUU
>hsa-novel-26a	UUUGUAGAAAUAAAUGUCCCUUU
>hsa-novel-26b	UUUGUAGAAAUAAAUGUCCCUUU
>hsa-novel-27	UAAGAACCGCAAUUACUUUUGC
>hsa-novel-28	CCAAAGUGAUUGUGGUUUUUGC
>hsa-novel-29	UAGUGGCCAGUGGCGAUCUCAUU
>hsa-novel-30	AAUCUCUUGAACCUGGGA
>hsa-novel-31	CAAGAACCGCAGUUACUUUUGC
>hsa-novel-32a	UGUGUGUGUGUGUGUGUGU
>hsa-novel-32b	UGUGUGUGUGUGUGUGUGU
>hsa-novel-33	AAAAACUGCAAUUUCCUUUGC
>hsa-novel-34	UUCUCACCACUGCACUUGACUAGUU
>hsa-novel-35	AAAACCGUGAUUACUUUUGC
>hsa-novel-36	UGCAAAAAUAAUUGUGGUUUU
>hsa-novel-37	CAAAGAGAAGCGAAGAUGGAGC

>hsa-novel-38	AAAGUGCUGGGAUUACA
>hsa-novel-39	AAAAACCGUAAUCACUUUUGCA
>hsa-novel-40a	AAAAGUAAUCGCGGUCUUUGCC
>hsa-novel-40b	AAAAGUAAUCGCGGUCUUUGCC
>hsa-novel-41	CGCGCCCGGCUCCGGGA
>hsa-novel-42	CCUGGCCAACAUGGUGAAACC
>hsa-novel-43	AAAAACCGCAAUGACUUUUG
>hsa-novel-44	UUCAGUUGUUCAACCAGUUACA
>hsa-novel-45	CCUGAGCCUUUCGAUUCGACC
>hsa-novel-46	UUCGAGACCAGCCUGGCC
>hsa-novel-47	UCCCUCUCCCUCCUUGCUCCUAC
>hsa-novel-48	CAAAAGCAAUUGCGGUUUUUGCC
>hsa-novel-49	UGGUAGAAAUCCAGAAUCUGAG
>hsa-novel-50	UUCGCUGGGAAUUCAGCCUCUGC
>hsa-novel-51	CCAUGGACAGGAUGGGCC
>hsa-novel-52	UCCAUAUCCCAACCUGUCAGAGU
>hsa-novel-53a	UGCACCCACUGUGAUAUUUGGA
>hsa-novel-53b	UGCACCCACUGUGAUAUUUGGA
>hsa-novel-54	UUUAUGAGAGAAGGUAUGACACU
>hsa-novel-55	GGCGGCGGCGGCGACUC
>hsa-novel-56	AAAGACCGCGAUUACUUUUGC
>hsa-novel-57	GAGGAUCACUUGAGCCCAGGAGUU
>hsa-novel-58	ACAAAGGUAAUUGUGGUUUUUGC
>hsa-novel-59a	CGGCGGCGGCGGCGGCG
>hsa-novel-59b	CGGCGGCGGCGGCGGCG
>hsa-novel-60a	CGGCGGCGGUGGCGGCG
>hsa-novel-60b	CGGCGGCGGUGGCGGCG
>hsa-novel-60c	CGGCGGCGGUGGCGGCG
>hsa-novel-60d	CGGCGGCGGUGGCGGCG
>hsa-novel-61a	CGGCGGCGGCGGCGGCGGC
>hsa-novel-61b	CGGCGGCGGCGGCGGCGGC
>hsa-novel-61c	CGGCGGCGGCGGCGGCGGC
>hsa-novel-61d	CGGCGGCGGCGGCGGCGGC
>hsa-novel-61e	CGGCGGCGGCGGCGGCGGC
>hsa-novel-61f	CGGCGGCGGCGGCGGCGGC
>hsa-novel-61g	CGGCGGCGGCGGCGGCGGC
>hsa-novel-61h	CGGCGGCGGCGGCGGCGGC
>hsa-novel-61i	CGGCGGCGGCGGCGGCGGC
>hsa-novel-61j	CGGCGGCGGCGGCGGCGGC
>hsa-novel-61k	CGGCGGCGGCGGCGGCGGC
>hsa-novel-61l	CGGCGGCGGCGGCGGCGGC
>hsa-novel-61m	CGGCGGCGGCGGCGGCGGC
>hsa-novel-61n	CGGCGGCGGCGGCGGCGGC
>hsa-novel-62	UGGCUGUGCCAUCCCAGGGACC
>hsa-novel-63	UUGGGCUCUUCUCCCAUCUGU
>hsa-novel-64	ACCCUCAGUCCGUAUUGGUCUCU
>hsa-novel-65	UAAAAGUAAUUGUGGUAUUUGC
>hsa-novel-66	GGAGGAUCACCUGAGGUCAGGAGUU
>hsa-novel-67a	GUAGGUGGCCUGACUGGC
>hsa-novel-67b	GUAGGUGGCCUGACUGGC
>hsa-novel-68	AACCAGGCAAGAACUACUGUCU
>hsa-novel-69	CCUGGGUGGUGGUGCAU
>hsa-novel-70	CAAAAGUAGUUGUGGUUUUUGCC
>hsa-novel-71	UCCCUGUCCUCCAGGAGCUCAC
>hsa-novel-72	UAUGUCUGCGGACCACCCACC
>hsa-novel-73	AUGUGCUUGGCUGAGGC
>hsa-novel-74	UAAAGCUGUAGCAUUAACCCCAU
>hsa-novel-75	AAAGGUAACUGCGGUUUUUGC

>hsa-novel-76	AAAAGUUAUUGUGGUUUUUGCU
>hsa-novel-77	GUGGAGAAAUAGUGGCUAAC
>hsa-novel-78	CCCUGUCCUCCAGGAGCUCA
>hsa-novel-79	CCUGUCUGAGCGUCACU
>hsa-novel-80	AGCCUGUCUGAGCGCCGCU
>hsa-novel-81	UGAGGUAGUAGGUGGUGUGC
>hsa-novel-82	AAAAGGCGGGAGAAGCCC
>hsa-novel-83	UCAGCCACCCGCCACUGCACCUG
>hsa-novel-84	ACCCCACUCCUGGUAGCU
>hsa-novel-85	ACAGGACAGUGAUGCCC
>hsa-novel-86	CCAGGUGCUGUAGGCUUU
>hsa-novel-87a	UCCCUGUCCUCCAGGAGCU
>hsa-novel-87b	UCCCUGUCCUCCAGGAGCU
>hsa-novel-87c	UCCCUGUCCUCCAGGAGCU
>hsa-novel-88a	CCUGGUGCUCCUGGUGCU
>hsa-novel-88b	CCUGGUGCUCCUGGUGCU
>hsa-novel-89	UAGGUUGUAGGAUGCUAAACAGA
>hsa-novel-90	AGGCUGGAGCUCAGGGA
>hsa-novel-91a	UGAGACUGGCCCUGCUGG
>hsa-novel-91b	UGAGACUGGCCCUGCUGG
>hsa-novel-92	AUCCUGGGUUUCGGCACCA
>hsa-novel-93	AUGUUACUAUAGGUAUUUUGCU
>hsa-novel-94a	CUAGUGGGCCACUUUUG
>hsa-novel-94b	CUAGUGGGCCACUUUUG
>hsa-novel-95	UGGCACCUGUGGUCCCAGCUACU
>hsa-novel-96	UAUUCAGUUAGUUCAGAAGAUCU
>hsa-novel-97a	GUGCCUGUAGUCCCAGC
>hsa-novel-97b	GUGCCUGUAGUCCCAGC
>hsa-novel-97c	GUGCCUGUAGUCCCAGC
>hsa-novel-97d	GUGCCUGUAGUCCCAGC
>hsa-novel-97e	GUGCCUGUAGUCCCAGC
>hsa-novel-97f	GUGCCUGUAGUCCCAGC
>hsa-novel-98	AAGCAGCACAGUAAUAUUGG
>hsa-novel-99	AUCAUAGAGUGUACUUACA
>hsa-novel-100a	CUCUCUCUCUCUCUCUC
>hsa-novel-100b	CUCUCUCUCUCUCUCUC
>hsa-novel-100c	CUCUCUCUCUCUCUCUC
>hsa-novel-101	ACUGAACUUGGAGUCAGAAGAC
>hsa-novel-102	CCAGGUGUUCUUUGAGCUUC
>hsa-novel-103	CGAGUGUGUGUGUGUGAGA
>hsa-novel-104	CCCUCUCUCUCCCUCUCCC
>hsa-novel-105	AAGGAGCUCACAGUCUAUUG
>hsa-novel-106	CAGGCACAGUGGCUCACGC
>hsa-novel-107	AAGGAGCUUGCAAUCUAGA
>hsa-novel-108a	CAGCCUCUGGCAUGGUG
>hsa-novel-108b	CAGCCUCUGGCAUGGUG
>hsa-novel-109	AACCUAGUCAGAUUAGUUCUC
>hsa-novel-110	AGGCUGGAGUGCAGUGGCU
>hsa-novel-111	UUGAUUUUUUGUUUCAGUCACU
>hsa-novel-112	CGAAAACUGCAAUUGCCUUUGC
>hsa-novel-113	CCCCUGGGCUGUUACUGUUCCC
>hsa-novel-114	UCACCUGGCAUAAGCAAUUCAC
>hsa-novel-115	UUAAGCACUCAAUACAUGAUAGU
>hsa-novel-116	UGAGUGUGUGUGUGCGAGUGUGU
>hsa-novel-117	AGCCACUGCACUCCAGC
>hsa-novel-118	CCUGAAAUCUGAACUGUGUUCA
>hsa-novel-119	UGUAAACAUCCUUGACU
>hsa-novel-120	CGCCUGUCUCAGCGUCC

>hsa-novel-121	CUGAAGGCGCUGGAUACAGU
>hsa-novel-122	AAGGAGCUCACAGUCUA
>hsa-novel-123	UGAGGUAGUGGUUUGUAAAGU
>hsa-novel-124	GUAAAACUGCAAUUACUUUUG
>hsa-novel-125	AAACACUGUCAGGUAAAGA
>hsa-novel-126	GCAAACGUAAUUGUGGUUUUU
>hsa-novel-127	CGUCGUCGUCGUCGUCG
>hsa-novel-128	UUCCCAGCCAAUGCACCA
>hsa-novel-129	CAGGAAUAGAGGAAACCAGCUU
>hsa-novel-130	GGCUGGUCCGAGUGCAGUGGUGUUU
>hsa-novel-131	UUCAGCCACCUGAGAUU
>hsa-novel-132	AACAACCCUAUCCUCUUCCAGA
>hsa-novel-133	AGGCUGGAGGAUCGCUU
>hsa-novel-134	GUGAAAUGUUUAUGACA
>hsa-novel-135	UGAGCUGGGCUGAGGUG
>hsa-novel-136	AAAAGAGAACUUUGAAG
>hsa-novel-137	ACAGCAGGCUCAGACAGGCAGG
>hsa-novel-138	AAGAGUAAUUGUGGUUUUGCCG
>hsa-novel-139	CGUGUCCAGAAUGGCCAGCCAG
>hsa-novel-140	CCUGGAAAGAAUGGAGA
>hsa-novel-141	UGCUACCCAGCACAGGGUGGUCCU
>hsa-novel-142	UUGGCUGGGAUGAUGAU
>hsa-novel-143	CCUGGCAGCCCUGGUGAA
>hsa-novel-144	CCCCACGUUGGGUGCUA
>hsa-novel-145	UUUCUGCGACCAAUCAGACU
>hsa-novel-146a	UCUCCUAUGAAGCAACCGCUGCA
>hsa-novel-146b	UCUCCUAUGAAGCAACCGCUGCA
>hsa-novel-147	GCUGGAGUGCAGUGGCUCGAU
>hsa-novel-148	UUAGAAGGGCUGGAAGAAGGCA
>hsa-novel-149a	ACAUUCCAGUGUGUUGUAACAU
>hsa-novel-149b	ACAUUCCAGUGUGUUGUAACAU
>hsa-novel-150	CCUAGCAGUCUCAGGACACACA
>hsa-novel-151	GUUCUCAGAGUUCCACCAGAUCA
>hsa-novel-152	UCCUGGGCUCAAGUGAUCCUC
>hsa-novel-153	CCUGGAACUCCUGGCCCUGU
>hsa-novel-154	UUAGGCACUCAAUACAUGAUAG
>hsa-novel-155	AGGGUGAACCUGGUCCU
>hsa-novel-156	GUGAAAUGUUUGGGACCAG
>hsa-novel-157	CUGACAAGCCAGGCUUCUGGUC
>hsa-novel-158	UCUGGAAGUUGAACACACCAC
>hsa-novel-159	GCAGUCUGCCUUUCUUU
>hsa-novel-160	CCCAGAAAUCUGGUAUGCCAGC
>hsa-novel-161	CAUCCUAGCUUGCCUGAGACU
>hsa-novel-162	AGGCAGUGAAGUUAGCUGAUGGC
>hsa-novel-163	CAGGCUAAAUUUUCCCGUCAUC
>hsa-novel-164	UUACAUUAAGAUUAGAGACAAGA
>hsa-novel-165	GAAAUGCAGUUUGCUUGGAGC
>hsa-novel-166	GCCUGGAGUUCCUGGAGA
>hsa-novel-167	AUUUUCCCCAUCUCCUGGAAGCC
>hsa-novel-168	UUAUCCUCCAGUAGACUAGGGAG
>hsa-novel-169	UUCCUCUUCUUAACACC
>hsa-novel-170	GAGGCUGAGGCACGAGAAUCACUU
>hsa-novel-171	GCUGUGUUAAUUCUCUUCUGUA
>hsa-novel-172	UGGCAUUCCCCUGCUAGGAUGG
>hsa-novel-173	UUCAGAGAUGUCUAAGAAAGUG
>hsa-novel-174	GCCCCGUCGGGCCCGGG
>hsa-novel-175	UGGCGUACGGGAGCCCCGC
>hsa-novel-176	UUCAGUGUCUGGUGAGGGUC

>hsa-novel-177a AGGCCCGGCGGGGAAGG
>hsa-novel-177b AGGCCCGGCGGGGAAGG
>hsa-novel-178 GAAAGUUUGAGGAACUACU
>hsa-novel-179 CGGCCCUGGCGGCGCGC
>hsa-novel-180 AGUGGGUUAUUGUUAAGA
>hsa-novel-181 CUCAGGACUGGGUCAUUCUGUUC
>hsa-novel-182 UGAGGCCGAGAAGGCAACCGCGA
>hsa-novel-183a CGGGGCUGGGCGCGCGC
>hsa-novel-183b CGGGGCUGGGCGCGCGC
>hsa-novel-184 CACGUGGCUCCUGCUUGUCAUC
>hsa-novel-185a CGGCGCGACCGCUCUCC
>hsa-novel-185b CGGCGCGACCGCUCUCC
>hsa-novel-186 GGAGCGGGGACAGCAGC
>hsa-novel-187 GCAAAAACCACAGUUACUUUUG
>hsa-novel-188 UAGGUCACUGGGGUCAGAGCC
>hsa-novel-189 UUCCUUGUACCUGGAUUGGCC
>hsa-novel-190 UGGUGGGCUGUCUCCCAGGAAGC
>hsa-novel-191 CUGUUGGGCUUGGCUCU
>hsa-novel-192 AUCGCUUGAGCCCAGGA
>hsa-novel-193 CAAAACCGCGAUGACUUUUGCAC
>hsa-novel-194a GGGGCGCGCGCCGCGGC
>hsa-novel-194b GGGGCGCGCGCCGCGGC
>hsa-novel-195 UUUGUCCCAGCUAGAUGAGUGC
>hsa-novel-196a CGCCGCGGCCGGCUCCGG
>hsa-novel-196b CGCCGCGGCCGGCUCCGG
>hsa-novel-197 GGGGACGGCUGGGGAGGCC
>hsa-novel-198 UGGAUGCCUCUUUCCCUGCAGG
>hsa-novel-199 CCUGGUCCUGCUGGUCC
>hsa-novel-200a GUGGCGGCGGCGGCGGC
>hsa-novel-200b GUGGCGGCGGCGGCGGC
>hsa-novel-201 UCAGGCUCAGUCCCCUCC
>hsa-novel-202 GGCGGCCUGUCCCCAGU
>hsa-novel-203 CUAGGCUCGCACUAGACUCAGC
>hsa-novel-204 CGGCGGCGGCGGUGGCGGC
>hsa-novel-205a UUCCAAAAUACUGGUUCUUU
>hsa-novel-205b UUCCAAAAUACUGGUUCUUU
>hsa-novel-206 UAGGAUACACUGGUUAAGCACU
>hsa-novel-207a AGGCAUUAGAUUCUCAUAAGGA
>hsa-novel-207b AGGCAUUAGAUUCUCAUAAGGA
>hsa-novel-207c AGGCAUUAGAUUCUCAUAAGGA
>hsa-novel-208 CGCCGCUUUCUGGGCUCGCUC
>hsa-novel-209 GAGGAGGCGCCGCCGCC
>hsa-novel-210 GUGGACUGUGUGAGGCA
>hsa-novel-211 UAGAGAACACAAGAAUACCUU
>hsa-novel-212 AGGCUGGACGAGGCGCC
>hsa-novel-213 CGGAACCUUAGAGCUUCAGC
>hsa-novel-214 CCUCCCGGACGGGGCGGCU
>hsa-novel-215 CGGCGGCGGCGGCGGGACC
>hsa-novel-216 UGCCUGGAGAAAGAGGA
>hsa-novel-217 AGAGAACCAUCUGAAAGACUUU
>hsa-novel-218 AGGCAGUAGGUUGUAUAGUUA
>hsa-novel-219 GUGCUUGGCUGAGGAGC
>hsa-novel-220 AGGCCCUGCUGGCUCCCC
>hsa-novel-221 CUGACUGAGGGACAGUG
>hsa-novel-222 GGCAUUGGCUGGCUUUGG
>hsa-novel-223 CAGGGGCCGGCGGCGGC
>hsa-novel-224 UUAGGGCCCUGGCUCCAUC
>hsa-novel-225 UCCGUGGGUGGUGGUAC

>hsa-novel-226a	CAAAAACUACAGUUACUUUUGC
>hsa-novel-226b	CAAAAACUACAGUUACUUUUGC
>hsa-novel-227a	CCAGGAGGCUGAGGCCC
>hsa-novel-227b	CCAGGAGGCUGAGGCCC
>hsa-novel-227c	CCAGGAGGCUGAGGCCC
>hsa-novel-228	AUCUGAGAGUACUUUGUCAAGG
>hsa-novel-229	GUGCACGUGAGGAUGGCUCUGA
>hsa-novel-230	CUGGCGCUUUCACACACUC
>hsa-novel-231a	UGCACUCCAGCCUGAGC
>hsa-novel-231b	UGCACUCCAGCCUGAGC
>hsa-novel-231c	UGCACUCCAGCCUGAGC
>hsa-novel-231d	UGCACUCCAGCCUGAGC
>hsa-novel-232	UCGCAGUUGGAUCUUGG
>hsa-novel-233	CGGGUGCUGUAGGCUUU
>hsa-novel-234	CUGCCGGGAGUUCCCUUCAAGC
>hsa-novel-235	CUUGGGAGCGGGCGCGC
>hsa-novel-236	CGGGGAGGCAGUGACGA
>hsa-novel-237	AUCACAUUGCCAGGCUUUA
>hsa-novel-238a	UGAGCAAGUGAAGUAUGUGGUA
>hsa-novel-238b	UGAGCAAGUGAAGUAUGUGGUA
>hsa-novel-239	GUGAACAGGUAAGAGCCC
>hsa-novel-240a	UCCCUGUUCGGGCGCCA
>hsa-novel-240b	UCCCUGUUCGGGCGCCA
>hsa-novel-241	CUGCCCCAGGUGCUGCUGAU
>hsa-novel-242	CCGAAACUGCAAUUACUUUUGC
>hsa-novel-243	CCUUCGCGCACUCUAACU
>hsa-novel-244	GGGUCUCCAAGGUGAAC
>hsa-novel-245	GCAACAUAGCGAGACCCGGUCUCUA
>hsa-novel-246a	CUGGAAAGGCGGCCGCC
>hsa-novel-246b	CUGGAAAGGCGGCCGCC
>hsa-novel-247	UCCCCGGCAUCACCACCA
>hsa-novel-248	CUGUUCCCAGUGCGCCC
>hsa-novel-249	AGGCUGGGCUGUGAUCCGC
>hsa-novel-250	CUGGCGGAGCGCUGAGG
>hsa-novel-251	AUAACUGAGCUUCCUCUUGCAGC
>hsa-novel-252	AGUUUGACUGGGGUGGU
>hsa-novel-253	UGUGCCUGGGACUCCACC
>hsa-novel-254	UCCACCCCUCCUCCCGC
>hsa-novel-255	AAAUCCAAAAUCUGAAA
>hsa-novel-256	GGUGCUGUGGGCACUGCU
>hsa-novel-257a	CUGUGAAUAGCCACUGCACU
>hsa-novel-257b	CUGUGAAUAGCCACUGCACU
>hsa-novel-258	CGCCUGUGAAUAGCCACU
>hsa-novel-259	UUGGCUGCAGUUAAGGUGG
>hsa-novel-260	AUCCCACUUCUGACACCA
>hsa-novel-261	CUGGGCUCAAGCAAUCC
>hsa-novel-262	UAAAGUAAUCGUGGGUUUUGCC
>hsa-novel-263	UUGCAGGCACUGGGAGA
>hsa-novel-264	AUUGAAUUUUUGUUUGUGACCU
>hsa-novel-265	GGCGGCGGUGGCGGCGGC
>hsa-novel-266	AGGCCCUGCUGGCUCCC
>hsa-novel-267	AUCUCGGACGAGCCCCCA
>hsa-novel-268	AAGUGAUUGCGAUUUUUGCC
>hsa-novel-269	CGGCAGCGGCGGCGGCGGC
>hsa-novel-270	UGAGGCAGUAGAUUGUAUU
>hsa-novel-271a	CGUGGGGAACCUGGUGCC
>hsa-novel-271b	CGUGGGGAACCUGGUGCC
>hsa-novel-272	CUCUGGAGCCUCUGGUCCU

>hsa-novel-273	AAACUCUAAGGUGACUUACCCU
>hsa-novel-274	UCAGGAGUUUGAGACCAGCC
>hsa-novel-275	UGAGUGUGCGUGUGUGAGUGUGC
>hsa-novel-276	AGCUACAUAUGGCUACUGAG
>hsa-novel-277	CGCGCCCCGGCCCCGCGUC
>hsa-novel-278	GUGAGGAGGCUGUGGCC
>hsa-novel-279	CAGACACAGGUAUGGCUGGCUCC
>hsa-novel-280	CGCCCCUCCCCGCCCCU
>hsa-novel-281	AUUUUUAUUGCCACAACU
>hsa-novel-282	GAAAUUCUUGGACAGGC
>hsa-novel-283	UGAGACCAGCCUGGCCAAC
>hsa-novel-284	UCAAGGAGUUCACAGUCUAGU
>hsa-novel-285	CGCCGCCCCUCCUCCUCC
>hsa-novel-286	CGUGGGGAACCUGGCGCU
>hsa-novel-287	ACCAGGAGGCAGAGGCCCU
>hsa-novel-288	AGGACCCAUUGGAGGGC
>hsa-novel-289	CGGGAGCUGUAGGCUUU
>hsa-novel-290	UGCCUCAGCUCAGCAGGAA
>hsa-novel-291a	AACACAGUGGCUAGGUUCUG
>hsa-novel-291b	AACACAGUGGCUAGGUUCUG
>hsa-novel-292	GGGACUGAUGGCUCAGCU
>hsa-novel-293	UGAGAUGCCCUUUCGAACAU
>hsa-novel-294	CUGGCCCUGCUGGUGCU
>hsa-novel-295	CGGGACGGCUGGGAGGGU
>hsa-novel-296	GCUGUAGCGAAGAGAGUU
>hsa-novel-297	CACCAGGCACAGACAGGCAA
>hsa-novel-298	UAUCUGCUGUUGUCCCCUCAGG
>hsa-novel-299	AGUGGGAGACCCUGACUCU
>hsa-novel-300	UCUGCUGGUGCUCCUGGC
>hsa-novel-301	UGAGAUGCCCUUUCGAACAUAC
>hsa-novel-302	AAAGCAGGACGGUGGCC
>hsa-novel-303	UCCUGGGGCUGCAGGUG
>hsa-novel-304	AACUGAGAGUGGAGUGCU
>hsa-novel-305	AACAAAUACAGGAAAGAGUUCU
>hsa-novel-306	GGCAGGAGAAUUGCUUG
>hsa-novel-307	CGGCCCGGGUUCGAUUC
>hsa-novel-308	CAUAUUAUUGAACUGAGCAUAU
>hsa-novel-309	UACCAUUAUCUAUGAGAACAGA
>hsa-novel-310	ACCCCACCACUGCCACCA
>hsa-novel-311	UGUGAUUUCUGCCCGGC
>hsa-novel-312	ACUGAUGAUGUGUUGGU
>hsa-novel-313	GCAGGAGGUGUCAGGAA
>hsa-novel-314	CAGGCUGGUCUCAAACU
>hsa-novel-315	UCCUGCUGACUACGCCA
>hsa-novel-316	UGAGUGUGUGUGCGAGUGUGU
>hsa-novel-317	GUGGACCUGGUGCUGCU
>hsa-novel-318	AUCACUUUGGCACCAACCUAAU
>hsa-novel-319	UGGCUCUAAGGGCGGGG
>hsa-novel-320	CAGCACAGAAAUAUUGG
>hsa-novel-321	AAAGACCGCAAUUACUUUUG
>hsa-novel-322	UCUACUUCAAAUUCCUCCCU
>hsa-novel-323	AAGGACUACAACUCCCAGUGUGC
>hsa-novel-324	ACUUGCUUGAGCCCAGGAA
>hsa-novel-325	CCUCACACGGGGCCCCA
>hsa-novel-326	CUGAUCUCGGGUGAUCCACCC
>hsa-novel-327	CCAGGUGCUGUAGGCUUC
>hsa-novel-328	CAUGGCUGCUGUAAUGUGUGCA
>hsa-novel-329	GCUCAGUCCAGCAGUAAC

>hsa-novel-330	UUGUUGUUGUUGUUGUU
>hsa-novel-331	GAGUUCUGGGCUGUAGUGC
>hsa-novel-332	UUUUUUGCUGGAACAUUUCUGG
>hsa-novel-333	CGCCUGUGAAUAGCCACUGCACU
>hsa-novel-334	AGGGGCUGCGGGCCGGGC
>hsa-novel-335	GAUAAAGACAUACCUGAG
>hsa-novel-336	CAGAACAGGCGCUGCGG
>hsa-novel-337	CCAGCACUGAGUCAAGUCAGGU
>hsa-novel-338a	CCUGGCCCCUCUGGAGC
>hsa-novel-338b	CCUGGCCCCUCUGGAGC
>hsa-novel-339	UGAGGUGGGAGGAUCGCUU
>hsa-novel-340	CAGCAGGCACAGCCAGGCC
>hsa-novel-341	UCUUGUAGCAGGUAUUUUCCCU
>hsa-novel-342	CAGAAGACAUCAGAUGGUAACUUUU
>hsa-novel-343a	CGCUGGGUUGGGGGGCCC
>hsa-novel-343b	CGCUGGGUUGGGGGGCCC
>hsa-novel-343c	CGCUGGGUUGGGGGGCCC
>hsa-novel-343d	CGCUGGGUUGGGGGGCCC
>hsa-novel-344	UAGGAGCUAUCAGAACUUAGUG
>hsa-novel-345a	GCCCUGGGGAGCCUGGUC
>hsa-novel-345b	GCCCUGGGGAGCCUGGUC
>hsa-novel-346	UUUUGUGUGUCAGGGUGCAGGUC
>hsa-novel-347	AGGCAAGAUGCUGGCAUU
>hsa-novel-348a	UUGAGAAUUAUGAUGUGCCUA
>hsa-novel-348b	UUGAGAAUUAUGAUGUGCCUA
>hsa-novel-349	GGGAUUACAGGCAUGAGCC
>hsa-novel-350	GCUCUGAGAAGACGGUC
>hsa-novel-351	AUCCCACUCCUCACACCAGA
>hsa-novel-352	UCCCUGACCUCCAGGAGCCCAC
>hsa-novel-353	UGAAGGGUUCCAUGUGG
>hsa-novel-354	GACAGCCUGGACUUCCC
>hsa-novel-355	UCUCUGGGCCUGUGUCUUGGU
>hsa-novel-356	CACCUGAGUUGUAAAAA
>hsa-novel-357	UGGCUGGGACCCGGCUCCUCGC
>hsa-novel-358	CCAGAGCAUGACAGAACU
>hsa-novel-359	CUGGCUGGCGGCUGCGCU
>hsa-novel-360	UCAGGCCAUGAUUAAGA
>hsa-novel-361	UGCUCCUUAUGAGAAUCUAAUGC
>hsa-novel-362	ACCAGGGCAAAUGUUUGUACUUU
>hsa-novel-363	UAAGGGGUCACCCGGUGUCCGUCC
>hsa-novel-364	ACAGAAGUCUGAACAUUG
>hsa-novel-365	UAGUCCCUUUGCACCCCUGUCC
>hsa-novel-366	UGACUUCUUAUUCUUUCCUGUGU
>hsa-novel-367	CCAGGCACUGGGAAUAUAGCA
>hsa-novel-368	UGAUAUUUACUUAAAUCCUGAGG
>hsa-novel-369	CAGGCUACACCCUAGACC
>hsa-novel-370	CUGGGAUUACAGGCAUG
>hsa-novel-371	UCCGGUUUAAAUGGGUCCGAUU
>hsa-novel-372	ACAGGGAGGAAAGAAACC
>hsa-novel-373	UUCACAGUGGCAAAGUUA
>hsa-novel-374	UAAGUCUCUUUAUAUGUACUGGA
>hsa-novel-375a	UUGGCCAAGCAAGGCAUCUGCC
>hsa-novel-375b	UUGGCCAAGCAAGGCAUCUGCC
>hsa-novel-376a	AUCCCACCGCUGCCAGC
>hsa-novel-376b	AUCCCACCGCUGCCAGC
>hsa-novel-377	GGCUGGAGUCUGGUGCCUGAA
>hsa-novel-378	UGGAGCAGGAAGCCCGC
>hsa-novel-379	UCUCCAGUCCUCUUUUUGCCCUCA

>hsa-novel-380	UUUUUUUGAGACAGAGUCUUG
>hsa-novel-381	AUCACAGUGGCAAAGUUC
>hsa-novel-382	AACACAAGUGUAUGAGCUCAUA
>hsa-novel-383	CAUCUGGGGACCUGUCA
>hsa-novel-384	CAGGACUGAUGCUCCAACCAUA
>hsa-novel-385	AUGUCUCUGUGGCGCAAU
>hsa-novel-386	UCAGUCCAUUAGAUGUGAGUGC
>hsa-novel-387	AGGGGUGGCUGCAGGGCC
>hsa-novel-388	UGGCUCAGCUCCAAUUCUGCCCACG
>hsa-novel-389	UCCUGAGAAUUCAUGCAAAAGA
>hsa-novel-390	GCUGAGCCUGUGGCUCU
>hsa-novel-391	AUGCAGCCGGAGCUCCGC
>hsa-novel-392	AAUCAUUCACGGAAAACACC
>hsa-novel-393	UCCCAAAGUGCUGGAAUU
>hsa-novel-394	UGGCUGUGGCUGAGGCUGAG
>hsa-novel-395	CCAGGACCUGCGGAGGCU
>hsa-novel-396	UAAAACUGCAAUCACUUUUGC
>hsa-novel-397	ACUUGGCAGAGAACAAU
>hsa-novel-398a	GCUGGCUGGCGUGGAGC
>hsa-novel-398b	GCUGGCUGGCGUGGAGC
>hsa-novel-399	UUCACAGUGGCUAAGCUA
>hsa-novel-400	CGGCGCCUGAGCGGACUGA
>hsa-novel-401	GAUGUGCCUGUUCUCUGGCAGA
>hsa-novel-402	UCCCUGUCCUUCAGGAGCUG
>hsa-novel-403	CAUCUUCCCAAGCCUCCUCCAUC
>hsa-novel-404	UCUAGAGGAAGUAGCAGUC
>hsa-novel-405	CUGCAAAGCUGAAACUA
>hsa-novel-406	CUUGAGGCUGUUUGUCAAGCC
>hsa-novel-407	CUAGCCAGGAUUCCCUCA
>hsa-novel-408	CUGGGAGGCGGUCGGUUCUGAGUC
>hsa-novel-409	CCCCGGGAGGGAGCCAUGUUUCUUA
>hsa-novel-410	AUUCCUAGGUAUUUUAUU
>hsa-novel-411	UGACAAAGUGAGACCCUG
>hsa-novel-412	CUCACUGUAGCAUCGAACACCU
>hsa-novel-413	ACAUGGCUCCCAGCGUGC
>hsa-novel-414	UGGCGUGGAGCCAGGCG
>hsa-novel-415	CAGGAGCUUACAAUCUAGA
>hsa-novel-416	UUGGCAAGGUGAAGAGA
>hsa-novel-417	UUUGUGACCUGGUCCCCUC
>hsa-novel-418	UUCUUUAAGAGGAUUAAUCU
>hsa-novel-419	CAUGGCUGCCCUCUGGCA
>hsa-novel-420	CUCUGCGGGCCCGAGGAGG
>hsa-novel-421	AGGGCUGGGCUGGGUGCC
>hsa-novel-422	AUGUCAUCAAUGAAAACU
>hsa-novel-423	UCCUUCCUAGCACUGCUAAGC
>hsa-novel-424	UCCUGGCUGUCUCGCCA
>hsa-novel-425	AGGGCUGAGAUAACAGGC
>hsa-novel-426	AUGUUCUUAGGCUCAACGGCAUC
>hsa-novel-427	AGGAAGGAGAGGAGAGA
>hsa-novel-428	CCAGGCACUAAAGAGACACUGG
>hsa-novel-429	UUGGGGUGCAUGGAGCUUCACU
>hsa-novel-430	UAGCUUAUCGGGCUGAUGU
>hsa-novel-431	CAGGGAGAAGCUGGCUGGGUGGUGG
>hsa-novel-432a	AGGACUGGAUGUCGGGCUGCAUG
>hsa-novel-432b	AGGACUGGAUGUCGGGCUGCAUG
>hsa-novel-433	GUGGAAGCGGCAGACUCAGC
>hsa-novel-434a	AUUGGCACACAUUGAACCCUU
>hsa-novel-434b	AUUGGCACACAUUGAACCCUU

>hsa-novel-435	UUGAACUGCUCUAUGCUACUGUU
>hsa-novel-436	GCCAAGUCCCCUCAUCUUUCU
>hsa-novel-437	CCAUCUACUUCAAAUUCC
>hsa-novel-438a	CGGCUCAGAACGCAGGGG
>hsa-novel-438b	CGGCUCAGAACGCAGGGG
>hsa-novel-439	UCGGGGCACUCGGGUCUUUGCU
>hsa-novel-440	AGCGGCAGCGGCGGUGGC
>hsa-novel-441	CACACACUGCAUUCUAGUGACU
>hsa-novel-442	UAGUGGAUGAAGCACUGUGUGC
>hsa-novel-443	GUGGGCCCCAGCGUAGCACCCA
>hsa-novel-444	GGUGGCACCGGUCAGGACCUUGG
>hsa-novel-445	UGUGGAUGCAAAGAAAUUU
>hsa-novel-446a	CAGGGACAUACUUAUAUGAGAA
>hsa-novel-446b	CAGGGACAUACUUAUAUGAGAA
>hsa-novel-447	AGAACACAUCUCAUUGACCAGA
>hsa-novel-448	CCAGGCUCUGCUCUGCA
>hsa-novel-449	ACCCUCCUCCUGCCUGACC
>hsa-novel-450	UAAGACCCUAUCUUCCCUGGCAG
>hsa-novel-451	AAGUGGAUGAGGACUUCCCAGA
>hsa-novel-452	AGCGGCAGCGGAGCUCUUGGGC
>hsa-novel-453	AGUGUUCAGGACACCUGGUGGGGG
>hsa-novel-454	CGGGACUGAAGGAGCCCCUGCU
>hsa-novel-455	GAAAGCAUCUGUGGAAUG
>hsa-novel-456	UGGAAAGAAAAACCAAGUCUGA
>hsa-novel-457	GGAUGGGUGGAUCAGUG
>hsa-novel-458	AAGCAGGCACAGCCAGGCAG
>hsa-novel-459	AGGCUGGGCCUAGGAAAC
>hsa-novel-460	UGGGGCUCCGAAGGUUUCUGCCUGC
>hsa-novel-461	CGGCUCACCUGUGUCUCUGCA
>hsa-novel-462	UAUGUAGAGAGCGUCUACCAGGU
>hsa-novel-463	AGGAAGAGUGCUGGGCCC
>hsa-novel-464	UGCCUGUGGCAUGUUACAGAUU
>hsa-novel-465	CCCCUGCCUGGCACUCUGU
>hsa-novel-466	CCAGGCCUGAGGACAUGAGG
>hsa-novel-467	CGCCCCCUCUCUCUCUCUCC
>hsa-novel-468	CUCCUGGGCCCUGGCCC
>hsa-novel-469	CUCUGCUGGUGCUCCUGG
>hsa-novel-470	CCUUGGGCCUGCAUCUGACUCC
>hsa-novel-471	UGAGUUAAUAUCGUAUGUGAGU
>hsa-novel-472	AUGAGUGGGCCAGGUACC
>hsa-novel-473	UCUCAUCCUCUCCCUGCAGUU
>hsa-novel-474	CCAGGCUCUUUCUAGCUGCUUU
>hsa-novel-475	CUGGGCUGAAGGUCUCACGCU
>hsa-novel-476	CACCUGGAAUUUCUAUGAUAU
>hsa-novel-477	GUGUGGCUGGGCCUUCCU
>hsa-novel-478	CUGGUCUCUGUCCCCAACC
>hsa-novel-479a	GAGCGGAGGCUGAGUCUC
>hsa-novel-479b	GAGCGGAGGCUGAGUCUC
>hsa-novel-480	GUCCCUGUUCAGGCGCCA
>hsa-novel-481	UGGGAAAGCUGGAUUGGAGCUGGCC
>hsa-novel-482	UCACCGGGACAGAACCAUAUGU
>hsa-novel-483	UGCCUCCCACCCCUUCCCCAGU
>hsa-novel-484	CUGUCCAUGGGCUUCUCUGAU
>hsa-novel-485	CCUCUCUGCCGCAGGCUGG
>hsa-novel-486	ACCGGAGCCCCGGGACUGG
>hsa-novel-487	CUGCCAUGCCUCCUGCCCUCAGC
>hsa-novel-488	CGUGCAGUCGGGACACAGAGCU
>hsa-novel-489	GCUGGGGGGCCUGUAGAGGUG

>hsa-novel-490a	AGCACACUGAUCACAUUUAUGC
>hsa-novel-490b	AGCACACUGAUCACAUUUAUGC
>hsa-novel-491	UUCCCCUCGGCCCUGCCCCAC
>hsa-novel-492	CUGCCUGUCGUUAUGGCUGCUGCC
>hsa-novel-493	CCAGCAGGUGCAGAAUUCCACA
>hsa-novel-494	UGGCAAACCAUUCCUAGAAU
>hsa-novel-495	UGCCCUCCUUGGGAAUCUAC
>hsa-novel-496	AUGACCACGGGCUGAGCC
>hsa-novel-497	CCCUUUGCCCAGGAUGCU
>hsa-novel-498	GGCUGAUGUCAUCCCGGGCUGCUC
>hsa-novel-499	UGGGGCCAGGCUGACUGC
>hsa-novel-500	AAGGACUGAGCCGCACCU
>hsa-novel-501	UUGUUGCCAUGGACUAGGGUUCA
>hsa-novel-502	AUUGGCACUGCACAUGAUUGC
>hsa-novel-503	GGAGCUGCCCAGAAACUUUUC
>hsa-novel-504	UAGUGCCUAAGUGAUAGGGUUGC
>hsa-novel-505	UGUGGCCCGGGCUGCAGGAAAGC
>hsa-novel-506	ACGCUGACUGUAGCCCUGACC
>hsa-novel-507	CCCUGAGACUCCACAGC
>hsa-novel-508	UGGUCUGCUUGUUUCAUCUCC
>hsa-novel-509	CCAGGAGAGCACUUUCCC
>hsa-novel-510	GCCCCGGGUCGCUGCCGC
>hsa-novel-511	GGGUGAGCCUUGAAGCC
>hsa-novel-512	UUCAUGUAUUGCCUCCCAGUA
>hsa-novel-513	UGCUGGUAGGCAAAGAGGACC
>hsa-novel-514	UUGGGGGAUCACUGUGGUCGU
>hsa-novel-515	UGUCACCCUGGAUCACCAGCACU
>hsa-novel-516	GGGGCUGGAAGAGGAAAGC
>hsa-novel-517	CUGUGGCCUCCCCUUCCUCCU
>hsa-novel-518	CCUGGCCCUGCUGGUGCU
>hsa-novel-519	UCUGGUCUGGCACUGUGA
>hsa-novel-520	GAUUACAGCAGCUGUCCUCUCGC
>hsa-novel-521	CACACUGGACUGCAGGGAUC
>hsa-novel-522	CGGGGACUUAUGAGCAGGUCGCG
>hsa-novel-523	UCCUGCCUCCCCACCCUGUGGU
>hsa-novel-524	UUUCUCUCUCCCUCUGGGACCAGA
>hsa-novel-525	CAGUUCUUCCUGCCCCUC
>hsa-novel-526	CCACUCCUGCCUCACCCCAGC
>hsa-novel-527	CUGGUGCUGCUGGCCCU
>hsa-novel-528	GCAGGAGUCGGCUCUGAG
>hsa-novel-529a	CCUGGACUCGGGAUUUAAC
>hsa-novel-529b	CCUGGACUCGGGAUUUAAC
>hsa-novel-530	UUUGGCACCAUUAAAAAGUAC
>hsa-novel-531a	CAGCCCUCAGUGCUGCUGCAC
>hsa-novel-531b	CAGCCCUCAGUGCUGCUGCAC
>hsa-novel-531c	CAGCCCUCAGUGCUGCUGCAC
>hsa-novel-531d	CAGCCCUCAGUGCUGCUGCAC
>hsa-novel-531e	CAGCCCUCAGUGCUGCUGCAC
>hsa-novel-532	AGAGGCUGGAGCUCAGG
>hsa-novel-533	GUGGGGGGACUCUAGACA
>hsa-novel-534	ACUGCUCAUGACAAGAAUUCA
>hsa-novel-535	CUCCUGGGCUGGAGGAGUCU
>hsa-novel-536	GCCCCGGCGCUGUAAACC
>hsa-novel-537	CUGCCUGUACAUGUGAACGUUC
>hsa-novel-538	GGAAGUGGAGAGCUGGGAC
>hsa-novel-539	CUGGGAUUGGGUGUGCCACAGU
>hsa-novel-540	UGCAAAAUUUAAGGUGACACCCACU
>hsa-novel-541a	ACCCUCUGGGCUGAGUUCCUU

>hsa-novel-541b	ACCCUCUGGGCUGAGUUCCUU
>hsa-novel-542	GCAGGUGCAGACCUUGG
>hsa-novel-543	CUGGGCUGAGGGUGGGCA
>hsa-novel-544	GAACCUGGAAGAGAUGGAGA
>hsa-novel-545	GCUGUGCCUGGGACUCCGCCC
>hsa-novel-546	AGGCAUGGAAAUGAGAA
>hsa-novel-547	CUUUGUGCGCUGAGGCGG
>hsa-novel-548	AAUGUAACUGACAUCUUUGGGA
>hsa-novel-549	GUCUAGCCCCUUCCUUUCUC
>hsa-novel-550	UAAGGCCAUCGUAAGUUGA
>hsa-novel-551	UUGAGUCACAAGCACAGAACC
>hsa-novel-552	AUGGCAUCAUCUGUGAAU
>hsa-novel-553	GGCCUGGGAGCUCCAUGUU
>hsa-novel-554	GAGGAUGGGGCCUUGGAGGA
>hsa-novel-555	UGGGGUCCUGUUUUCUGGUCGU
>hsa-novel-556a	GCAGCAGGUGACAGGAGC
>hsa-novel-556b	GCAGCAGGUGACAGGAGC
>hsa-novel-556c	GCAGCAGGUGACAGGAGC
>hsa-novel-556d	GCAGCAGGUGACAGGAGC
>hsa-novel-557a	AGGGGCUGGUGUGCAGCU
>hsa-novel-557b	AGGGGCUGGUGUGCAGCU
>hsa-novel-558	UGCCUGGACCCACGAAGC
>hsa-novel-559	CCUGGUCCCUUCUCACUGU
>hsa-novel-560	CAGGGACAUGGGGCUGGG
>hsa-novel-561	UGUGGUUCUACUGACUCUCU
>hsa-novel-562	GCCCUGGGGAGCCUGGCCA
>hsa-novel-563	CUGUUGAAGCACCUCUGUCUUU
>hsa-novel-564	UACCCUGUAGAUCCCAAU
>hsa-novel-565	GGGGCUCAGAUGCUUCCUCUCC
>hsa-novel-566	UGGGGUGGGACUGAGGGA
>hsa-novel-567	CUAGUGGGUAGCAGAGUG
>hsa-novel-568	UCUGGGGUAGGGUGGCAGCGUCC
>hsa-novel-569	AAGGGACAUGGGUGGAAU
>hsa-novel-570	UCCUGCUGCAGAGGCCCU
>hsa-novel-571	AUGGGACAAUUUUGGAGCGCAU
>hsa-novel-572	CUUUCCCUGGAGACCUUGGUG
>hsa-novel-573	UGAGAACUGAAUUCCAAGUG
>hsa-novel-574	UCCUUCUGCUUCAGCCU
>hsa-novel-575	UCACCCUGACUAUGACCAGCAGCCC
>hsa-novel-576	UGAAAGGGCUCUGAGAUG
>hsa-novel-577	UCUGGCCUGGCUCUGUCGGC
>hsa-novel-578a	UCCCUGAAUUCAGCAAAUAUGU
>hsa-novel-578b	UCCCUGAAUUCAGCAAAUAUGU
>hsa-novel-579	CCUGGCUGUCCUCCUUCU
>hsa-novel-580	CAGGGCAUGCCAAGGGAG
>hsa-novel-581	AGGGCCGAGGAGCAUCUG
>hsa-novel-582	GUCUGUCUCCCUUGACUCC
>hsa-novel-583	CUCAGCAGACAAAAAAG
>hsa-novel-584	CUGCGAGGAGGUCGCACCAGC
>hsa-novel-585	CUCUGCCUGCCAGCCUGCC
>hsa-novel-586	UGGACUAGUCAUGAAGGAGCGGU
>hsa-novel-587	CACUCCAGGUGGUGGUGUGACU
>hsa-novel-588	AAUGGUUCCGUGAUUCAUGAAAAC
>hsa-novel-589	UAGGCAGUGUCUCUCCCCAGCU
>hsa-novel-590	CACUCGUGGCACUGUCAGC
>hsa-novel-591	UGUGUCACUGAGAACCACGUUCA
>hsa-novel-592	UCUGUGCCUCUUUUCUUUG
>hsa-novel-593	CUGAGGGAAUUCUGAAGCUG

>hsa-novel-594	AACUGGCCUUGGAACCUGUAGU
>hsa-novel-595	CUGUACAACUCACCAGCUGC
>hsa-novel-596	GUUCUGCCUGGCACUUGUGUGA
>hsa-novel-597	UGUCACUCUUGUGUGUUGCAG
>hsa-novel-598	AUGGACUGAGAGCCGUCUGCCCU
>hsa-novel-599	GGAUGGAUAGGCAGAUAGAUCU
>hsa-novel-600	CUGCCCUGGAGUUCUGUG
>hsa-novel-601	AUUUUUCCAAAUAAUUACCAUU
>hsa-novel-602	UUCCAGAGCCUGACUGCC
>hsa-novel-603	UUGGGGAGCACUUGUCC
>hsa-novel-604	UCCUGGGCCCCAUCCCAGAGUUCU
>hsa-novel-605	AAGUGAAUUGUAGAGGUC
>hsa-novel-606	AACGGUGACACUAAUUUCUGGU
>hsa-novel-607	AAGGGCAGGACAGGGGCC
>hsa-novel-608	GCUGGGGGGCUGCAGACAGCU
>hsa-novel-609	GAGCAGGGCGGUCCUCGC
>hsa-novel-610	UCUGUGGGCAGAUCAGCU
>hsa-novel-611	CCAGGUGCCCUUGGGUCUC
>hsa-novel-612	GCCGUCUCCAGGACCCAGCU
>hsa-novel-613	GCCAGCAGCUCCUACUUCC
>hsa-novel-614	GGACCUGGUGCACAGUAGG
>hsa-novel-615	AGAGGGAGAGGAGGGGCU
>hsa-novel-616a	CCUUGGAGACGGCCGCAGACACA
>hsa-novel-616b	CCUUGGAGACGGCCGCAGACACA
>hsa-novel-617	GUGAGUGUGGAGGAGCUUAAGA
>hsa-novel-618	UGGGACUGCCCAGGGCCAGG
>hsa-novel-619	CACUGUGGCUCGGGUCCC
>hsa-novel-620	UCUCAGCUCCAGAAUCCUCAGC
>hsa-novel-621	UGGCCCUGCUGGCCCUGCUG
>hsa-novel-622	GCCUGGAGCUGGGACGGCGCC
>hsa-novel-623	AUGAGCGAGACCUAGACACUUGGCA
>hsa-novel-624	AUGGGGUGAGGUGCAGAGAGA
>hsa-novel-625	CCACUGCCAGGCUGCUCUAUGGGAG
>hsa-novel-626	UCCUGCCUGGCUCUCCA
>hsa-novel-627	CUGGCUCUGGCAGGGUGC
>hsa-novel-628	AGGAUGACUCCUAGAUUCUUUC
>hsa-novel-629	UGCACUAAGUAUAUGCCAAGC
>hsa-novel-630	AACUUGGACCUCUGGGA
>hsa-novel-631	GGUGGGGGAACAGGUAAGC
>hsa-novel-632	GUGGCCCUGUCACUGGCUCU
>hsa-novel-633	AGAGGCCGGGCUGCAAGGCCA
>hsa-novel-634	UUGCAGCCAGGGAGCUUCCGGGC
>hsa-novel-635	UGGCUCAGUUCAGCUGGG
>hsa-novel-636	CUGGGACAGAACCCCACU
>hsa-novel-637	AAGUUCUGUAGAAAUGUUUGCA
>hsa-novel-638	GAGAGUCUCUGCUUCCCC
>hsa-novel-639	AUAGGAGGAGCAAACGUAGCACU
>hsa-novel-640	CUUGAGUCCAGGAGUUC
>hsa-novel-641	GCACCUGGGCCAGGGCCUG
>hsa-novel-642	CCGAGGAGCUGAAGAGUAGCAUUU
>hsa-novel-643	UAAAGUUUACUGCAUAGUGCU
>hsa-novel-644	UAGAGGAAGUAGCAGUC
>hsa-novel-645a	ACUCUCUGAGACCUUGGAAUA
>hsa-novel-645b	ACUCUCUGAGACCUUGGAAUA
>hsa-novel-645c	ACUCUCUGAGACCUUGGAAUA
>hsa-novel-646	GACAGUGUCUGAGAGAA
>hsa-novel-647	UGGCAGUGCAUACUGUUGGCU
>hsa-novel-648	UCCUUCUGUGCUGUGGUCCAGG

>hsa-novel-649	GUCCUUCUCGGAAUGUUCUUUG
>hsa-novel-650	CGCCCCACAUUCUGGGUCAAGCAUG
>hsa-novel-651	UCUAGGAGCUCACAGUCUAGG
>hsa-novel-652	AAAGGAGAAUUCACAUUU
>hsa-novel-653	CAGGGCUGUUUCGAGGC
>hsa-novel-654	CUCUCCUCUGCACCUGAA
>hsa-novel-655	CAGCAGGGCAGCACGGUGUCCAGGC
>hsa-novel-656	GAAUGUCAGAACCAGCCAGC
>hsa-novel-657	CUGAGCCACUCUGCCCACAG
>hsa-novel-658	UUGACUGACUGAACCGCCUCCU
>hsa-novel-659	AGCAGGACAGAAGAAGCC
>hsa-novel-660	AUUGGUCCUCCUGGCCC
>hsa-novel-661	CACACGGGGCACUGGCUGACUC
>hsa-novel-662	UGAGGAUGUGUUGUUGCU
>hsa-novel-663	AGGUGGAGCUCUGUGUGAGACU
>hsa-novel-664	AGCUUCCAGAGGACGCUGGA
>hsa-novel-665	GUGAAAUGCUUAGGACCAGA
>hsa-novel-666	UUCUCUGGCCUCUUCACC
>hsa-novel-667	GGGGCUGGUGGGCUAGGAGUCAG
>hsa-novel-668	AUUGCCAUGGACUCACCA
>hsa-novel-669	GUGAGGACCCGGGGCUCCUUUCUAC
>hsa-novel-670a	AGGGAGCUGAUUGGAAGU
>hsa-novel-670b	AGGGAGCUGAUUGGAAGU
>hsa-novel-671	GGGGUAGAACCCAAUCCCCC
>hsa-novel-672	AGGCUCUACCCCCACCUCU
>hsa-novel-673	GAGAGAGGAGCUGGGCCCUGAGACA
>hsa-novel-674	AUGGCCACUGCUCACUGCAGA
>hsa-novel-675	UCUGACACAUUCGUUUU
>hsa-novel-676	UUUCUCCCUGUCUGGCCCC
>hsa-novel-677	CCACCCAGGAACCUGUGU
>hsa-novel-678	GCUGCAACGCGAGGCUCAGG
>hsa-novel-679	UGGAGCCACAGGUGACC
>hsa-novel-680	GCUGGUCUGGGUGCUGCU
>hsa-novel-681	CCAGGUGUCUUCCAUUCUGUCC
>hsa-novel-682	CCUGAGGUGGGCUCUUCUCUU
>hsa-novel-683	CCUUGGAGGUAGAGAGUGC
>hsa-novel-684	UCUGCUCAGCCUGUGGGGACA
>hsa-novel-685	AGGGAGGAACACCUGAUC
>hsa-novel-686	UGGGAAGGGCUUUGAGGGA
>hsa-novel-687	GGAGGAACUGUGGCUACCCU
>hsa-novel-688	CAGAACAGCCUGCUCUGCC
>hsa-novel-689	AGCUGGGGCUGUGCAGCC
>hsa-novel-690	AGGCCCAAGAGGACCAGCUCA
>hsa-novel-691	GCCCCCGGGCGGCCUCGA
>hsa-novel-692	GGCUGGGGCUAAGGGCUG
