# Supplementary material for: sncRNA changes induced by tension in hypertrophic scar: sncRNAs in hypertrophic scar
Source: Acta Biochim Biophys Sin (Shanghai). 2022 Aug 5;54(8):1197–200. doi: 10.3724/abbs.2022103 (PMC9827810; doi:10.3724/abbs.2022103)
Supplement: 090_supplementary_figures [file 090_supplementary_figures.pdf]

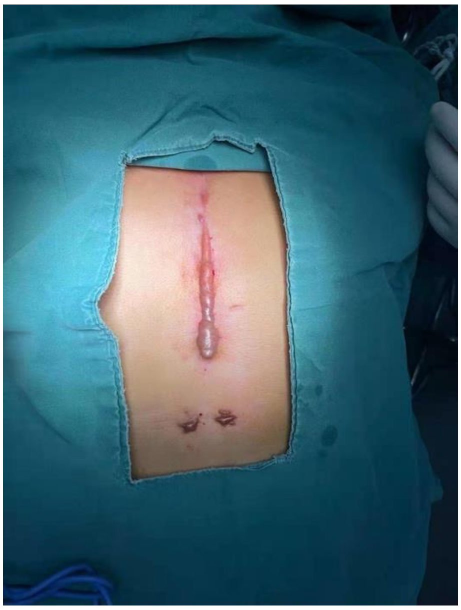

Supplementary Figure S1. The preoperative photo of the hypertrophic scar

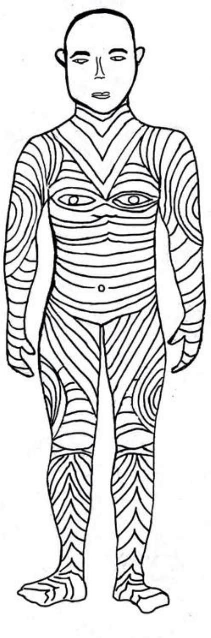

Supplementary Figure S2. Demarcation line of tension at the sternum skin

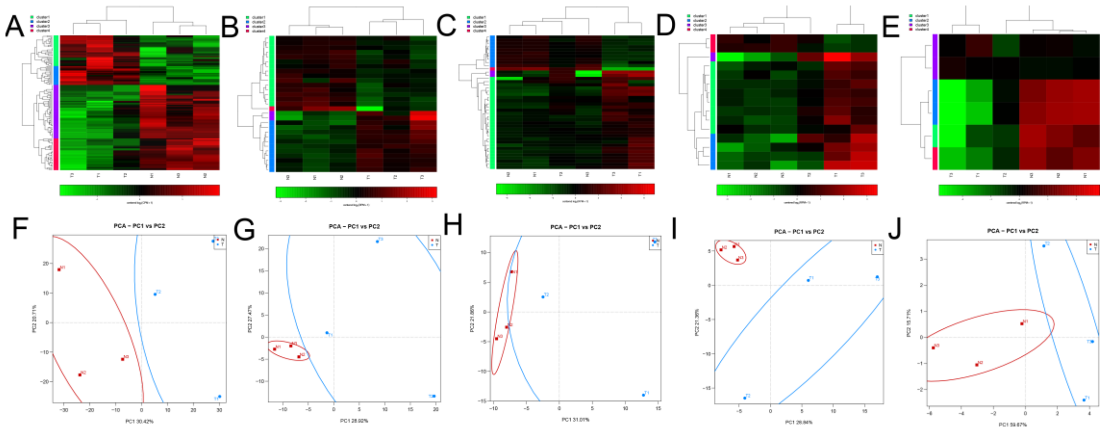

Supplementary Figure S3. sncRNA expression in hypertrophic scar with different tension (A–E) Heat map showing the differentially-expressed miRNA, piRNA, snoRNA, snRNA, repeatRNA in hypertrophic scar with different tension. (F–J) PCA analysis of miRNA, piRNA, snoRNA, snRNA, repeatRNA in hypertrophic scar with different tension. Data are shown as the mean±SD. **P*<0.05, and ***P*<0.01.
